# Motivated attention and task relevance in the processing of cross-modally associated faces: Behavioral and electrophysiological evidence

**DOI:** 10.3758/s13415-023-01112-5

**Published:** 2023-06-23

**Authors:** Annika Ziereis, Anne Schacht

**Affiliations:** Department for Cognition, Emotion and Behavior, Affective Neuroscience and Psychophysiology Laboratory, Georg-August-University of Göttingen, Goßlerstraße 14, 37073 Göttingen, Germany

**Keywords:** Associative learning, Faces, ERPs, Attention, Cross-modal

## Abstract

It has repeatedly been shown that visually presented stimuli can gain additional relevance by their association with affective stimuli. Studies have shown effects of associated affect in event-related potentials (ERP) like the early posterior negativity (EPN), late positive complex (LPC), and even earlier components as the P1 or N170. However, findings are mixed as to the extent associated affect requires directed attention to the emotional quality of a stimulus and which ERP components are sensitive to task instructions during retrieval. In this preregistered study (https://osf.io/ts4pb), we tested cross-modal associations of vocal affect-bursts (positive, negative, neutral) to faces displaying neutral expressions in a flash-card-like learning task, in which participants studied face-voice pairs and learned to correctly assign them to each other. In the subsequent EEG test session, we applied both an implicit (“old-new”) and explicit (“valence-classification”) task to investigate whether the behavior at retrieval and neurophysiological activation of the affect-based associations were dependent on the type of motivated attention. We collected behavioral and neurophysiological data from 40 participants who reached the preregistered learning criterium. Results showed EPN effects of associated negative valence after learning and independent of the task. In contrast, modulations of later stages (LPC) by positive and negative associated valence were restricted to the explicit, i.e., valence-classification, task. These findings highlight the importance of the task at different processing stages and show that cross-modal affect can successfully be associated to faces.

**Supplementary information:**

The online version contains supplementary material available at 10.3758/s13415-023-01112-5.

The human brain navigates the complexities of our everyday social lives very efficiently, for example, by quickly extracting various information from other people’s faces (Haxby et al., 2000). Research has repeatedly shown that what we know about a person and what is relevant to us impacts how we perceive that person (Bublatzky et al., 2014; Davis et al., 2009; Heisz & Shedden, 2009; Wieser & Brosch, 2012). This includes, but is not limited to, biographical information and relevant experiences with that person. In the laboratory, relevance often is manipulated through associations with valence-laden stimuli and actions, ranging from receiving monetary (Hammerschmidt et al., 2017, 2018a, b) or social reward and punishment (Aguado et al., 2012; Wieser et al., 2014) to highly aversive stimuli such as loud noise bursts (Watters et al., 2018) or electric shocks (Rehbein et al., 2014). It has been repeatedly shown that various types of affective stimuli impact face processing promptly (Wieser & Brosch, 2012) and through learned associations (Miskovic & Keil, 2012).

Although the term *attention* is not clearly defined in the literature, there is consensus that certain stimuli are processed preferentially over others, because they are physically salient, they resemble targets that match our current goals, or because we have learned their relevance through past experience. Especially experience-driven attention (Anderson et al., 2021) aims to explain phenomena, such as impaired performance in the presence of learned aversive distractors (Öhman et al., 2001; Vuilleumier, 2005) or self-referential cues as described in the cocktail-party effect (Röer & Cowan, 2021). To date, there is more evidence for conditioning effects with threat-related stimuli. However, also appetitive cues have been shown to be associated to different types of stimuli, e.g., faces, objects, or abstract stimuli, such as meaningless words (Aguado et al., 2012; Blechert et al., 2016; Davis et al., 2009; Hammerschmidt et al., 2017, 2018a, b; Rossi et al., 2017; Steinberg et al., 2013; Ventura-Bort et al., 2016). Although recognizing and reacting to both appetitive and aversive environmental cues appears adaptive, the need to detect and respond quickly is greater in a threatening environment (Öhman et al., 2001). Avoiding predictable and unpleasant situations also may be preferred over detecting potentially pleasant ones (Gottfried et al., 2002).

Neurophysiological research allows to investigate processes beyond overt behavior and has demonstrated that some acquired associations with affective stimuli elicit differential neural responses, even when a conditioned behavioral or physiological response has extinguished (Antov et al., 2020; Apergis-Schoute et al., 2014). However, in the case of absent effects in behavioral and neural measures, it remains open whether the information was learned at all, or whether it was not apparent under the specific test condition.

The overarching goal of the present study was to investigate how directed, experience-driven attention through task requirements impacts face perception at different levels (i.e., early/automatic vs. later/elaborate processing). More specifically, we tested whether the retrieval of valence-implicit or valence-explicit features moderates the neurophysiological and behavioral response to valence-based associations in faces.

Several affect-sensitive ERPs have been related to different stages of the processing of associated faces: The P1 typically peaks around 100 ms after face onset with an occipital, bilateral positivity. It is generated by the extrastriate cortex (Hillyard & Anllo-Vento, 1998; Russo, 2003) and has been reported to be enhanced for faces associated with affect-laden or valent stimuli, such as monetary reward (Hammerschmidt et al., 2017), emotional expressions of the associated face (Aguado et al., 2012), and threatening stimuli, although some fear-conditioning studies reported even earlier effects (Steinberg et al., 2013). More reliably than for the P1, associated and conditioned effects have been reported for the N170 and subsequent components. The N170 is a face-sensitive neural marker in the form of a negative deflection peaking around 170 ms over occipito-temporal regions, generated to a large extent by the fusiform face area (Gao et al., 2019). N170 effects of conditioned faces have been reported by a number of studies on fear-conditioning (Bruchmann et al., 2021; Camfield et al., 2016; Schellhaas et al., 2020; Sperl et al., 2021) as well as by studies on associated person knowledge (Luo et al., 2016; Schindler et al., 2021) and on conditioned facial expressions (Aguado et al., 2012). Modulations of the early posterior negativity (EPN), a relative negativity over occipito-temporal regions related to the early detection of emotional relevance and most pronounced around 200-300 ms for face stimuli, also have been reported for conditioned faces with different types of unconditioned stimuli (US), e.g., in conditioned fear (Bruchmann et al., 2021; de Sá et al., 2018; Schellhaas et al., 2020), and verbal descriptions about a person (Luo et al., 2016; Suess et al., 2014; Xu et al., 2016) and produced by a person (Wieser et al., 2014). Sustained motivated attention has been related to the late positive complex (LPC). Effects on the LPC, a centro-parietal positivity, have been reported for faces associated with different contexts in fear-conditioning (Bruchmann et al., 2021; Panitz et al., 2015; Rehbein et al., 2018; de Sá et al., 2018; Sperl et al., 2021; Wiemer et al., 2021), reward (Hammerschmidt et al., 2018b), and person-knowledge studies (Abdel Rahman, 2011; Baum et al., 2020; Kissler & Strehlow, 2017; Xu et al., 2016). Whereas there are several studies on cross-modal perception that include faces and affective voices (de Gelder & Vroomen, 2000; Pell et al., 2022), to our knowledge, the processing of faces, which have previously been associated with both positive and negative affect bursts, has not yet been tested.

The relevance of information to a specific situational task or context has been shown to play an important role in both learning and in retrieval (Shin et al., 2020). In learning, task-relevant (and thus context-congruent) information is supposed to be more easily integrated into a preactivated schema (van Kesteren et al., 2010). However, the relationship between task-relevance and context for retrieval is not straightforward, considering reports of generalized effects across different tasks. Similar to the processing of faces with emotional expressions (Hudson et al., 2021; Rellecke et al., 2012a; Schindler et al., 2020; Valdés-Conroy et al., 2014) and affective stimuli in general (Olofsson & Polich, 2008), task requirements are likely to moderate conditioned effects especially at later stages of processing (Schupp et al., 2006). That early and late effects have been reported in (not necessarily the same) fear-conditioning studies may be primarily caused by the use of intense and highly arousing stimuli. Additionally, most fear-conditioning studies have implemented valence and arousal ratings of the conditioned stimuli (CS faces) before and after the conditioning phase (Panitz et al., 2015; Rehbein et al., 2018; de Sá et al., 2018; Sperl et al., 2021), which might influence the attentional processes in other tasks, i.e., during learning and retrieval. Previously target-defining features of a stimulus have been reported to automatically withdraw processing resources even when they are no longer task-relevant (Kyllingsbæk et al., 2014). Nevertheless, conditioned effects have been reported for valence-unrelated tasks, such as old-new categorizations of faces (e.g., early effects: Hammerschmidt et al., 2017; late effects: Abdel Rahman, 2011; Baum et al., 2020; Kissler & Strehlow, 2017) and passive-viewing tasks (Xu et al., 2016).

Only a few studies systematically have investigated the role of attention on the perception of faces associated with context information. Three recent studies tested the effects of feature- and memory-based attention with different tasks, which included a) discrimination of lines that overlayed the faces, b) the faces’ gender (Bruchmann et al., 2021; Schindler et al., 2022) or age (Schindler et al., 2021), and c) the associated CS category. In their threat-conditioning study, Schindler et al. (2022) reported interactions between task and conditioning for the P1 and the EPN, but not for the N170 and LPC components and hence show no clear distinction between task influences on early and later processing. In contrast, associated verbal descriptions of crime-related actions differentially moderated early and late processing in Schindler et al. (2021), of which the N170 was enhanced in all tasks for negatively associated faces, whereas both associated effects on the EPN and LPC were reported only for the valence-focused condition. In these studies, associated context information was also presented during the test, either interspersed (in 33% of trials in Bruchmann et al., 2021; Schindler et al., 2022) or at the beginning of each task (Schindler et al., 2021). In experimental studies, researchers have often used intense and highly aversive stimuli to maximize the differentiability between conditions. While this is valid and important to demonstrate a general potential of associating context with faces, it neglects the real and true diversity of affective contexts, especially positive associations. Moreover, it has not been particularly well researched whether associating affective stimuli of lower intensity and whose contextual relevance has not been made explicit for learning also elicit robust effects in face perception. Expressions in faces and voices naturally co-occur and construct a perception of the whole person (Freeman & Ambady, 2011). Emotional expressions in both modalities are situation-dependent and naturally vary within individuals; conversely, facial and vocal expressions share inherent social and biological relevance (Straube et al., 2011). Both factors might impact the effectiveness of using these stimuli in associative learning, making them a compelling research topic.

## Goal of the study

To address this gap, we investigated the role of attentional focus in the retrieval of faces associated with cross-modal affect. To do so, we used a valence-implicit (old-new) and valence-explicit (valence-classification of the associated voices) task in a delayed test session to investigate associated valence under different attention conditions while recording face-sensitive ERPs. For learning, we paired faces displaying neutral expressions with short auditory affect bursts of positive (elation and amusement), negative (anger and disgust), and neutral (yawning and throat-clearing) valence, because they rapidly unfold emotional information and do not have the segmental structure of speech. In our newly developed Internet-based learning phase, unlike in classical (Pavlovian) or instrumental learning paradigms, our participants studied the face-voice pairs to correctly assign them to each other (similar to learning with flashcards). In addition, we did not provide any further information about the task requirements of the test session to not prompt participants to pay attention to specific stimulus features.

## Hypotheses

Our global hypothesis was that task requirements during the test would activate (goal-directed) memory-based attention to the associated face-voice pairs, which in turn would modulate the processing of the faces. More specifically, we expected the differential effects of task on different processing stages of valence-based associations, with early processing being less impacted than later, more elaborate processing (according to Rellecke et al., 2012a). In this sense, goal-directed attention through the task and experience-based attention through the relevance association would produce additive effects on visually-evoked potentials.

### Learning

In our online learning hub, participants could study the face-voice pairs flexibly and according to their own schedule. As a result, we expected high variability in the individual learning styles and in the time it took to reach the predefined learning criterion (95% correct in 24 subsequent test trials), and analyzed the learning data only in an exploratory way.

### Test: Behavioral hypotheses

We expected an effect of task difficulty with slower responses and lower accuracy for the valence-classification task (3-choice responses) compared to the old-new-task (2-choice responses). Furthermore, we expected an interaction effect of task $\times$ valence with larger RT and accuracy differences between the affectively and neutrally associated faces for the valence-classification task compared with the old-new task, as the latter required only superficial recognition of the faces. Regarding the valence effect in the valence-classification task, we expected higher accuracy for affectively compared to neutrally associated faces. Furthermore, faces previously associated with voices expressing an emotion of positive valence (i.e., elation and amusement) should be rated as more likable than faces associated with neutral bursts, and analogously, bursts of negative emotions (i.e., anger and disgust) should be rated as less likable compared to neutral bursts (similar to but not as pronounced as in Suess et al., 2014).

### Test: ERP hypotheses

We expected visually evoked potentials related to face perception to be modulated by the emotional valence of the associated voices, such that individual expressions of emotion would cluster according to their valence (e.g., amusement and elation for positive valence). Additionally, we expected modulatory effects of task, and interaction effects of task $\times$ valence, especially on the mid- (EPN) and long- (LPC) latency ERPs. However, for comparability with other studies and due to inconsistent findings on the influence of goal-directed attention (via task demands), we tested the interaction (task $\times$ valence) in all of our models. We predicted larger (mean and peak) amplitudes of the P1 for affectively (i.e., positively or negatively) associated faces than for neutrally associated faces and similar effects in both tasks. Although we expected valence-based modulations of the N170 and EPN, we did not specify the direction of the effects, as effects of associated valence have been inconsistently reported for these components. In contrast to the P1 and N170, we expected that EPN differences between affectively and neutrally associated faces would be more pronounced in the valence-classification task. The EPN is suggested to reflect enhanced sensory encoding of valence-laden stimuli (independent of the task). However, it is unknown whether recently conditioned faces would also produce an EPN component modulated by the associated valence in a superficial task like the old-new task (e.g., Rellecke et al., 2012a for reduced emotion effects on the EPN for facial expressions in superficial tasks). Finally, we did not expect effects of associated valence on the LPC due to unreported effects in similar studies. However, in the case of effects of associated valence, we expected them to be exclusive to the valence-classification task.

## Method and materials

This study was preregistered on https://osf.io/ts4pb.

### Participants

Of the 61 participants, who signed up for the study, 54 started learning, and 43 completed the EEG test session, of which our target sample size of 40 participants met the required number of trials (min. 30 valid EEG trials per valence condition and task after artifact rejection). Our sample consisted mainly of students (38 of 40; 30 females, 10 males, 0 diverse; age: 18-32, *M* = 21.62 years) who reported normal or corrected-to-normal vision (max. ±1 diopter), normal hearing, and no neurological or psychiatric disorders. All participants were right-handed, according to Oldfield (1971), and proficient in German. We recruited via campus advertisements and postings on social media, the university’s job portal, the website of the Institute for Psychology, and the department’s recruitment database. Participants were reimbursed with a fixed amount of money for completing the online learning phase and an additional hourly rate for the test session in the laboratory, or an equivalent amount of course credits.

### Stimuli

Twenty-four faces were selected from the Goettingen Faces Database (Kulke et al., 2017) and presented in their natural color on a light gray background. The face stimuli were edited and combined with a transparency mask that covered the hairline, ears, and neck. In the test session, they had a visual angle of approx. 3.2 $\times$ 4.8 degrees and a resolution of 200 $\times$ 300 pixels. The mean luminance (HSV) of the images ranged from 0.45 to 0.48 (*M* = .47; ${\upchi }^{2}$(528) = 550, *p* = .246) (Dal Ben, 2019). Affect bursts (happy, elated, angry, and disgusted) were selected from a validated database (Cowen et al., 2019) and supplemented with neutral vocalizations (clearing throat, yawning) from the social media platform “youtube.” All silent periods at the beginning and the end of the sound files were manually trimmed and normalized to −23 LUCS (Loudness Units Full Scale) using the open-source software Audacity® (v.2.4.2, Audacity Team, 2021). The perceived loudness of the audio files was normalized based on an algorithm following the EBU R 128 recommendation (https://tech.ebu.ch/docs/r/r128.pdf) for limiting the loudness of audio signals. Compared to other normalization methods (peak and RMS normalization), this method resulted in the smallest range of estimated loudness across stimuli using the R package Soundgen (Anikin, 2019). There were two separate stimulus sets of faces. One set included the 12 CS^+^ faces used in the learning and test phases, and the other set contained 12 new faces, which were used in the old-new task and the rating task of the test session. The assignment of the CS^+^ faces to the US voices was counterbalanced and matched for gender. Each emotion category (positive: amusement and elation, neutral: yawning and throat-clearing, negative: disgust and anger) of the voices entailed two stimuli in our experiment (one female and one male). Hence, each valence category contained four stimuli (four positive, four negative, and four neutral), resulting in a total of 12 face-voice pairs included in the learning phase. There were six different versions of the learning set for the face-voice pairs. Participants were pseudo-randomly assigned to one of the six versions to ensure a balanced distribution of stimulus-set versions.

### Procedure

**Fig. 1:**
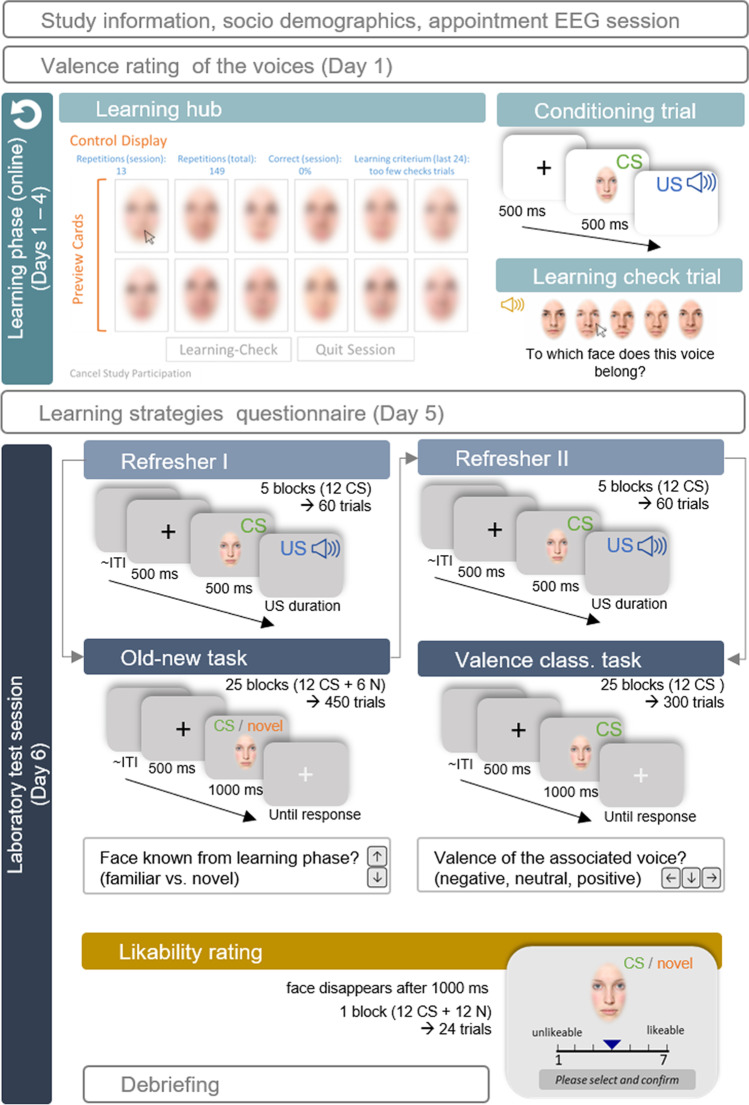
Procedure of the online learning phase and test session

The study was conducted in accordance with the Declaration of Helsinki and approved by the local ethics committee of the Institute of Psychology at the University of Göttingen. Before participation, interested participants visited a website that informed them of the complete procedure, inclusion criteria, data policy, corona regulations, and remuneration for the online learning phase and the EEG session. They were redirected to a form to provide contact and socio-demographic information if they consented. We scheduled EEG sessions with eligible participants and created personalized links and participant codes for the learning platform (learning hub). The link was activated six days before the scheduled EEG session. To participate in the EEG testing in the laboratory, participants had to achieve a learning criterion (95% correct out of 24 test trials) during one of the learning sessions and, independently of that, complete obligatory learning checks during the first four days. Participants were free to choose the length and number of learning sessions, repetitions, and learning checks within the learning phase. An overview of the learning and test procedure is shown in Fig. 1.

### Learning phase (online)

The online experiment was programmed in JavaScript with self-written and existing functions from the open-source library jsPsych (v6.3.1, de Leeuw, 2014). The experiment was integrated into JATOS (v3.5.8, Lange et al., 2015) on a local server installation at the University of Goettingen for data management. Participants could start the learning sessions with a personalized link and a participation code. Instructions were given compulsorily at the beginning and optionally displayed for later sessions. When participants logged in for the first time, they rated the valence of the auditory stimuli: On two sliders (without initial thumb), they were asked to rate 1) how positive vs. negative the mood of the speaker expressing the vocalization was, and 2) how pleasant vs. unpleasant they found the auditory stimulus. Then, regardless of their ratings, we individually presented all auditory stimuli with emotion labels to set an anchor for stimuli that may have been ambiguous for the participant. Subsequently, participants were redirected to the learning hub, where they could start their first learning session.

During the learning (association) phase, participants aimed to learn the pairing of the 12 face-voice stimuli (i.e., which face belonged to which voice) within 4 days by using the learning hub. The main page consisted of 12 preview cards showing blurred versions of the 12 faces. Clicking on one of the blurred cards started a conditioning trial with a central fixation cross, followed by the unblurred CS^+^ face and the auditory US starting with the face offset. The number of conditioning trials per CS-US pair was recorded for each session and in total. To assess whether they were able to allocate the face-voice pairs, participants were required to complete at least one obligatory learning check (including 24 test trials, approx. 2 minutes) per day. A learning check trial consisted of a pseudo-randomly selected US voice, played while participants had to select the correct face out of five gender-matching faces. Immediate feedback on the correctness of their response was provided. The learning criterion was met if 95% of the last 24 trials within a session were answered correctly. To prevent early and late learners from having different time delays between learning and test session, we required daily learning checks independent of the learning criterium. On top of the learning deck, information about the number of repetitions (conditioning trials) per session and in total, as well as the accuracy in learning checks for the session and the last 24 learning checks, was displayed. The order of the preview cards was shuffled at the beginning of each new session. For learning checks, a list of all face-voice pairs was shuffled, and the number of test trials was sampled from this list without replacement. The order of faces to choose from in the learning checks was also randomized. Participants could cancel their participation in the online study at any time and request the deletion of their data. Once participation was canceled, it was impossible to resume or restore the data or to participate in the EEG test session.

Questionnaire about learning strategies: One day before the test session, participants completed a questionnaire about their strategies for learning the face-voice pairs. Participants who reached the learning criterium were informed in more detail about the procedure and the safety regulations and were asked for their confirmation of the test session.

### Test session

After giving written consent, participants were prepared for the EEG session and seated in a dimly lit, electrically shielded room in front of a computer screen at a distance of approximately 78 cm. Two loudspeakers were placed to the left and right of the monitor. Participants positioned their chins in a height-adjustable chin rest. For the presentation of the laboratory experiment, along with standard Python (2.7) libraries, such as numpy and scipy, we used functions of PsychoPy (Peirce, 2009) for the presentation of the faces, PyGame (Shinners, 2011) as the audio library, and PyGaze (Dalmaijer et al., 2013) for the communication with the eye-tracker. After a welcoming message, the eye tracker was calibrated with a 9-point calibration. For all participants, the test session had the same order: Refresher trials I (5 $\times$ 12 trials), Old-new task (25 $\times$ 18 trials), refresher trials II (5 $\times$ 12 trials), valence-classification task (25 $\times$ 12 trials), and a likability rating (24 trials). To not reveal that any emotion- or valence-relevant task would be part of the experiment, the specific task instructions were shown before each task. Refresher trials were passive-viewing trials in which participants did not have to respond. However, we instructed them to focus on the face-voice pairs to “refresh” what they had learned. For the other tasks, specific instructions were followed by four example trials using face-like shapes and the correct answer as a label on top to familiarize with the response keys. Only here did participants receive feedback on whether they were correct and had the possibility to clarify the remaining questions with the experimenter. Breaks for stretching and relaxing were scheduled between tasks, and there were four additional breaks within the old-new task and three within the valence-classification task. A drift correction of the eye-tracker (1-point calibration) was implemented to resume or start the next task.

In all tasks, the order of faces or face-sound combinations was shuffled at the beginning of each block, with each block consisting of a single set of associated faces (or all 12 associated faces plus six randomly selected faces from the set of novel faces). Assignments of response keys were counterbalanced. Participants were instructed to answer as accurately and fast as possible, and to guess if unsure. Refresher trials started with a black fixation cross in the center of the screen for 500 ms, which was replaced by one of the CS^+^ faces displayed for 500 ms. With the offset of the face, the US set in (duration varied between stimuli). After a jittered inter-trial interval (*M* = 2,800 ms, *SD* = 200 ms), the subsequent trial began. In the **old/new task**, the participant’s task was to decide whether a face was known from the online learning phase or a novel set of faces. A black fixation cross was displayed for 500 ms in the center of the screen replaced by either a CS^+^ face from the learning phase or a CS^-^ face (novel). All faces were presented individually for 1000 ms and participants could respond as soon as the face stimulus set in. With the offset of the face, a gray fixation cross was displayed if no answer had been registered yet and continued until an answer was given via keypress. After the face-offset and a registered response, the next trial started after an additional jittered inter-trial interval (*M* = 1,800 ms, *SD* = 200 ms). In the **valence-classification task**, participants had to recall the valence category (negative, positive, neutral) of the associated voices, with only the CS^+^ faces presented. The presentation duration of the trial elements (fixation cross, face, response fixation cross, inter-trial interval) was identical to the old-new task. At the end of the test session, participants rated the likability of the CS^+^ and the novel CS^-^ faces. The faces were presented individually for 1,000 ms and were rated on a Likert scale in the appearance of a 1-7 slider positioned below the faces. There was no value or choice shown by default. A value was selected by clicking on the slider with the mouse, but had to be confirmed to start the next trial. At the very end of the session, participants were informed about the main aims and background of the study (presented on the computer screen) and could clarify any questions with the experimenter.

### Collected data

For each learning session, the number of conditioning trials for each individual face-voice pair and the accuracy of the learning check trials were recorded. In the test session, in addition to performance (RT and accuracy), we recorded EEG and pupil size (see Supplementary Information) during the refresher, old-new, and valence-classification tasks. No neurophysiological measures were collected during the likability rating.

### EEG recording and preprocessing

The continuous EEG was recorded with a sampling rate of 512 Hz (bandwidth: 102.4 Hz) at 64 active electrodes (AgAgCl) mounted in an electrode cap (Easy Cap™). The arrangement was based on the extended 10-20 system (Pivik et al., 1993). Additionally, two external electrodes were used: one each for the left and right mastoids. Reference electrodes were the common mode sense (CMS) active electrode and, as ground electrode, the driven right leg (DLR) passive electrode. The scalp voltage signals were amplified by a BiosemiActiveTwo AD-Box and recorded with the software ActiView. The data were preprocessed offline in MATLAB (2018) with functions of the toolbox EEGLAB (2019.9, Delorme & Makeig, 2004). To account for a systematic delay that was measured with a photodiode, event markers were shifted by a constant of 26 ms. The continuous data was re-referenced to average reference (excl. external electrodes), filtered with a 0.01 Hz second-order Butterworth filter, and the remaining 50-Hz line-noise was corrected with a function of the plugin “CleanLine” (v1.04, Mullen, 2012). Before performing independent component analysis (ICA), data was epoched from −500 ms to 1,000 ms around face-onset and the mean of the prestimulus baseline (−500 ms to 0 ms) was subtracted. Extended Infomax ICA was performed after a PCA reduction to 63 channels on a 1-Hz high-pass filtered copy of this dataset. The resulting ICA weights were transferred to the original 0.01-Hz filtered dataset. Independent components (ICs) were removed if labeled as muscle (>80%), eye (>90%), and channel-noise (>90%) components using “IClabel” (v1.2.4, Pion-Tonachini et al., 2017). Remaining diverging channels (>3 SD) were spherically interpolated. Then, epochs were trimmed to −200 to 800 ms and baseline-corrected (−200 ms to 0 ms). Trial-wise artifact rejection was performed: amplitudes exceeding −100/100 $\mu$ V (*M* = 42.15; 4.84%), steep amplitude changes (> 100 $\mu$ V within an epoch; *M* = 3.80; 0.44%), improbable activation (>3 SD of the mean distribution for every time point; *M* = 108.33; 12.4%) were excluded. Overall, the mean rejection rate was 15.29%. Eye blinks during baseline or face presentation were excluded in a separate step using pupil data. We extracted the following ERPs based on time windows and regions of interest (ROI) electrodes of a previous study (Ziereis and Schacht, in revision): mean and peak amplitudes for the P1 (80-120 ms) at an occipital electrode cluster (O1, O2, and Oz); mean (and peak)^1^ amplitudes for the N170 (130-200 ms) at an occipitotemporal electrode cluster (P10, P9, PO8, PO7); mean amplitudes for the EPN (250-300 ms) at an occipitotemporal cluster (O1, O2, P9, P10, PO7, and PO8); and mean amplitudes for the LPC (400-600 ms) at an occipito-parietal electrode cluster (Pz, POz, PO3, and PO4). In addition, we explored ERP effects between familiar and novel identities in the old-new task. We analyzed a mid-frontal FN400 (300-500 ms, at Fc3, F3, Fc4, F4), which has been related to familiarity of faces (Curran & Hancock, 2007), and a later parietal old/new component LPON (500 800 ms at CP1, CP2, P3, and P4), which has been related to episodic memory and recollection (Proverbio et al., 2019). Notably, the later old/new component overlaps in time and topography with the LPC component. We also explored auditory processing of the refresher trials in voice-locked N1-P2 ERP complex with N1 (90-145 ms) and P2 (165-300 ms), both with the identical frontocentral electrode cluster: F3, F1, Fz, F2, F4, FC1, FC3, FC2, FC4, C3, C1, Cz, C2, C4, CP1, CP3, CPz, CP2, and CP4, which we have included in the Supplementary Information.

### Statistical analysis

Tables with statistical models (incl. estimates, confidence intervals, stability measures, and likelihood ratio tests) are in the Supplementary information. All statistical analysis was conducted in R (v 4.0, R Core Team, 2020). All statistical models but the beta inflated distribution model (see below) were sum-contrast-coded, reflecting main effects rather than marginal effects. Here, the intercept corresponds to the (unweighted) grand mean, and lower-level effects are estimated at the level of the grand mean. The significance of the predictors was tested with likelihood ratio tests (LRT) of models including the predictor against reduced models and a null model. Post-hoc contrasts were used to test the difference between factor levels using “emmeans” (Lenth, 2020). We used the conventional significance level $\alpha$ = .05 (two-sided) and for posthoc tests Šidák-correction to adjust for multiple comparisons. To estimate the parameters in the analyses, we used the maximum likelihood (ML) estimator. For the 95% confidence intervals we used nonparametric bootstrapping (nsim = 999) if not specified otherwise.

#### Ratings of the voices

Due to the nature of the slider response measure with lower and upper bounds, we used a beta inflated distribution model (GAMLSS family “BEINF”; Stasinopoulos & Rigby, 2007) for the ratings of the voice stimuli. The model allows zero and one as values for the response variable. The beta inflated distribution is given as

$$\begin{array}{ll}f\left(y\right)={p}_{0},& \mathrm{if \;}(\mathrm{y}=0),\\ f\left(y\right)={p}_{1},& \mathrm{if\; }(\mathrm{y}=1),\\ f\left(y|\alpha ,\beta \right)=\frac{1}{B\left(\alpha ,\beta \right)}{y}^{\alpha -1}{\left(1-y\right)}^{\beta -1}& \mathrm{otherwise}\end{array}$$

for y = (0,1). The full model included the predictors emotion of the voice stimulus, type of the rating (valence of the voice vs. personal reaction to the voice), their interaction, and a random intercept for participant ID.

#### Learning phase (learning speed)

We modelled accuracy of the learning check trials until the learning criterion was reached (for the first time) with a binomial mixed model (GLMM). Predictors of the binomial mixed model were valence, number of learning checks (per valence), and their interaction. We included random slopes of valence and number of checks and the random intercept participant ID.

#### Test session

Only correctly answered trials were included in the ERP analysis. The study had a 2 (task: old-new/valence-classification) $\times$ 3 (valence: negative/positive/neutral) within-subjects design. For all outcomes (P1, N170, EPN and LPC amplitudes), mixed models with the fixed effects valence (positive, negative, neutral), task (old-new and valence-classification), their interaction, and the random effect (intercept) participant ID were analyzed. Although we expected the associated effects to reflect valence rather than the individual emotion categories, we included models for all ERPs with the fixed effects task and emotion (6 levels) and their interaction. In addition to these ERPs, for the old-new task, we analyzed the FN400 and LPON in a separate models and added the level (“novel”) to the predictor variables valence/emotion.

For response time data, only correct trials were selected. Separately for each participant, task, and condition, data were trimmed to a maximum cutoff of 5,000 ms after face onset and a skewness-adjusted boxplot method was used to exclude extreme values (function “adjbox” of the package “robustbase,” Maechler et al., 2021; based on Hubert & Vandervieren, 2008). After averaging across participants and conditions, response time data still resulted in skewed residuals. By taking the natural log of the averaged response times, the distribution of residuals became less skewed. We reported all model parameters on the log scale. Our model included valence, task, the valence $\times$ task interaction as fixed effects, valence and task as random slopes and participant ID as random intercept. The model allowed random slopes and the random intercept to be correlated. Additionally, for the old-new task, we tested for response time differences between familiar and novel faces in a separate model by adding the level (“novel”) to the predictor variable valence.

We ran mixed logistic regression models (binomial GLMMs) on the accuracy data of the test session. The predictor variables (UVs) were task (old-new and valence-classification), valence of the associated sounds (negative, neutral, positive), and their interaction. Because we detected overdispersion in the preregistered model (which included only participant ID as a random intercept), we maximized the random effects structure for the model including valence with a random intercept of participant ID, a random slope for valence and a random slope for task. Including a slope for the interaction between valence and task resulted in singularity issues and was dropped from the model.

For the likability rating, we ran ordinal-mixed-models with valence (including novel) or emotion as fixed effects and random intercepts for face and participant ID. Model estimates and their 95% confidence intervals are reported as odds ratios.

## Results

### Learning session

#### Valence rating of the voices

**Fig. 2:**
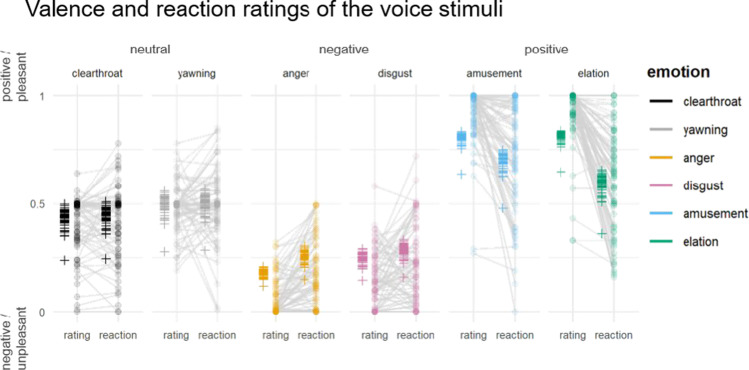
Rating of the vocal bursts (pre-learning). Stimuli were rated regarding the speakers’ emotional valence (positive/negative) and the personal reaction to the stimuli (unpleasant/pleasant) on separate sliders without an initial thumb. Dots represent the raw rating data per stimulus and participant. Crosses represent the predicted values per participant based on the zero-one-inflated GAMLSS model.

Before the first learning session, participants evaluated the individual voices along the dimensions “valence of the speaker’s expression” and “reaction to the burst.” The zero-one-inflated model showed a main effect of emotion (${\upchi }^{2}$(0.70) = 6, *p* = .008), rating type (valence rating vs. reaction: ${\upchi }^{2}$(23.10) = 828.21, *p* < .001) and a significant emotion $\times$ rating type interaction (${\upchi }^{2}$(6.34) = 137.78, *p* < .001). Consistent with the prespecified valence categories, participants rated the bursts as negative, neutral, and positive vocal expressions. However, their overall personal reaction to the stimuli was more homogeneous across emotion categories, with less positive reactions to elation ($\beta$_elation_reaction_ = −1.48, CI = [−1.85; −1.12]) and amusement ($\beta$_amusement_reaction_ = −0.84, CI = [−1.20; −0.48]) and less negative reactions to anger ($\beta$_anger_reaction_ = 0.67, CI = [0.30; 1.04]); Fig. 2.

#### Repetitions of face-voice pairs

The number of repetitions of face-voice pairs varied across participants. The total number of repetitions ranging from 18 to 428 and, for a given valence group, from 4 to 7 to 125 to 157. When proportions were considered, positive face-voice pairs were repeated least frequently (median = 32%) but had the largest range (25-47%), followed by neutral (33%; 22-41%) and negative (35%; 26-45%) face-voice pairs.

#### Learning speed (accuracy) by valence

**Fig. 3:**
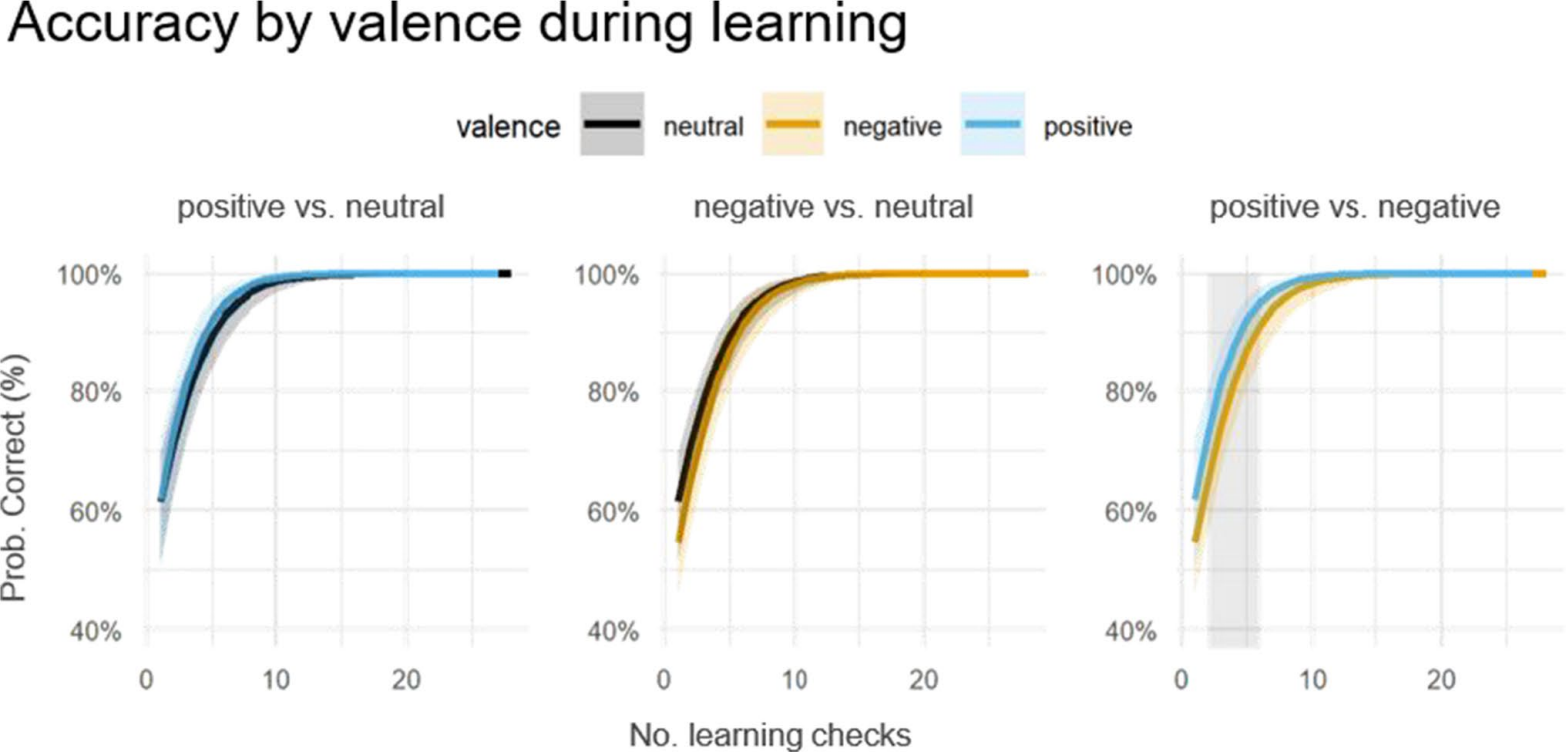
Predicted accuracy for learning checks. The x-axis refers to the number of learning checks separately per valence category and the y-axis to the predicted probability of a correct answer in a learning check. The light-gray highlighted area reflects significant differences between valence categories (predicted accuracy of one curve outside the 95% CI of the other).

There was a main effect of number of learning checks (${\upchi }^{2}$(1) = 51.09, *p* < .001), no effect of valence (${\upchi }^{2}$(2) = 1.9, *p* = .387), but a valence $\times$ check number interaction (${\chi }^{2}$(2) = 7.95, *p* = .019). Until the learning criterion was met, there were differences in learning speed between valence categories. Positive face-voice pairs were learned significantly faster than negative face-voice pairs at early check trials (predicted accuracies were outside 95% point-wise CI of the other valence category between the second and sixth learning checks per valence category). Differences between other valence categories over time were not significant (Fig. 3).

#### Learning strategies

Except for the mandatory learning checks, the learning phase could be organized flexibly by the participants. To gain more knowledge about how they experienced learning (i.e., perceived difficulty and subjective learning styles), we asked all participants to complete an online questionnaire the day before the lab session. Overall, participants varied in how difficult they rated the learning task. On a Likert scale ranging from 1 (very hard) to 5 (very easy), studying the face-voice pairs and reaching the learning criterion were rated on average as rather easy (*M* = 3.76, SD = 0.85). All participants indicated that certain face-voice pairs were more difficult to memorize. However, participants differed in what they specified as difficult: high similarity between faces (*n* = 23), lack of distinctive facial features (*n* = 16), gender of the face (female faces easier (*n* = 3), male faces easier (*n* = 5)), emotion (neutral more difficult (*n* = 4), anger and disgust within male faces more difficult (*n* = 1), and subjective mismatch between faces and voices (*n* = 4).

The majority of participants (*n* = 33) indicated that they used at least one specific strategy to study the face voice pairs, of which mnemonic device^2^ (*n* = 28) was mentioned most often, followed by focusing on specific distinctive facial features (*n* = 26) in order to be able to distinguish faces. Less frequently, they reported that they formed sub-groups of stimuli (e.g., female pairs first) and learned them separately (*n* = 5). Most participants began by using the card deck (*n* = 25). However, after a while some (*n* = 6) preferred to use mainly the learning checks to look at the faces for a longer duration and to get feedback on which faces still needed practice. Only two participants initially used the spatial information of the preview cards but stopped because the positions were shuffled in each session.

Participants rated their everyday ability to memorize faces on a 5-point Likert scale from 1 (very hard) to 5 (very easy) as rather high (*M* = 3.92, SD = 1.10). This self-reported ability did not significantly correlate with the number of learning checks required to meet the learning criterion (*r*(36) = −0.24, *p* = .814).

### Test session

Table 1 contains the averaged means and standard deviations of the behavioral measures of and ERPs of the test session.

#### ERP results

##### **P1:** Associated valence

P1 mean amplitudes were neither modulated by task (${\upchi }^{2}$(1) = 0.46, *p* = .497) nor valence (${\upchi }^{2}$(2) = 0.14, *p* = .931) nor their interaction (${\upchi }^{2}$(2) = 0.54, *p* = .764). Similarly, P1 peak amplitudes were neither modulated by task (${\upchi }^{2}$(1) = 0.67, *p* = .413) nor valence (${\upchi }^{2}$(2) = 0.22, *p* = .896) nor their interaction (${\upchi }^{2}$(2) = 0.91, *p* = .635).

##### Associated emotion

Replacing valence with the individual emotion categories did not change the results of P1 mean amplitudes (task:${\upchi }^{2}$(1) = 0.26, *p* = .607; emotion: ${\upchi }^{2}$(5) = 2.07, *p* = .839; task $\times$ emotion: ${\upchi }^{2}$(5) = 0.96, *p* = .965). Similarly, a model including emotion did not significantly explain P1 peak amplitudes (task: ${\upchi }^{2}$(1) = 0.99, *p* = .319; emotion: ${\upchi }^{2}$(5) = 5.33, *p* = .377; task $\times$ emotion: ${\upchi }^{2}$(5) = 1.41, *p* = .923) (Table 1).

**Table 1.** Mean (SD) accuracy (%), RT (ms), ERP amplitudes in μV of the test session for valence and emotion by task

		Neutral			Positive			Negative			Novel
Task	Measure	Avr. neu	Yawning	Clear throat	Avr. pos	Elation	Amusement	Avr. neg	Disgust	Anger	Novel
Old/new task	Acc.Test	98.6 (4.04)	99.1 (1)	98.1 (7.69)	98.83 (3.23)	99.45 (1)	98.2 (6.19)	98.35 (3.17)	98.25 (4.53)	98.45 (3.72)	98.45 (1.76)
	RT	725 (136)	726 (149)	723 (121)	722 (139)	721 (137)	723 (146)	733 (144)	743 (164)	724 (133)	756 (116)
	P1	4.05 (4.22)	4.02 (4.32)	4.04 (4.25)	4.08 (4.08)	3.98 (4.06)	4.17 (4.25)	4.08 (4.05)	4.16 (3.96)	3.93 (4.17)	
	P1 peak	7.04 (4.15)	7.2 (4.36)	7.51 (4.16)	6.93 (3.80)	7.06 (3.90)	7.4 (3.94)	6.88 (3.89)	7.32 (3.80)	7.1 (4.05)	
	N170	-6.73 (3.78)	-6.87 (4.01)	-6.55 (3.74)	-6.61 (3.90)	-6.75 (3.82)	-6.49 (4.04)	-6.74 (3.85)	-6.51 (3.91)	-7.05 (3.86)	
	N170 peak	-10.73 (4.57)	-11.14 (4.69)	-10.97 (4.55)	-10.7 (4.68)	-10.97 (4.62)	-10.96 (4.80)	-10.88 (4.56)	-10.93 (4.60)	-11 (4.54)	
	EPN	-2.08 (3.30)	-2.21 (3.48)	-1.91 (3.31)	-2.19 (3.25)	-2.38 (3.11)	-2.03 (3.50)	-2.51 (2.97)	-2.33 (3.23)	-2.73 (2.89)	
	LPC	5.32 (3.07)	5.25 (3.04)	5.36 (3.25)	5.25 (3.45)	5.21 (3.52)	5.28 (3.51)	5.24 (3.19)	5.39 (3.60)	5.1 (2.91)	
Valence-classification task	Acc.Test	95.38 (9.50)	96.2 (9.11)	94.55 (11.84)	97.08 (4.58)	97.05 (6.23)	97.1 (5.51)	96.7 (6.09)	97 (8.66)	96.4 (8.46)	
	RT	957 (148)	943 (152)	974 (167)	930 (143)	919 (122)	944 (187)	934 (178)	928 (182)	945 (202)	
	P1	4.08 (4.41)	3.97 (4.43)	4.14 (4.46)	3.96 (4.18)	3.98 (3.92)	4 (4.60)	3.98 (4.01)	4.02 (3.96)	3.91 (4.20)	
	P1 peak	6.81 (4.26)	7.07 (4.24)	7.34 (4.26)	6.91 (3.99)	7.2 (3.67)	7.2 (4.46)	6.86 (4.08)	7.12 (4.02)	7.05 (4.35)	
	N170	-7.04 (3.70)	-7.14 (3.76)	-6.99 (3.75)	-7.15 (3.76)	-7.2 (3.79)	-7.12 (3.82)	-7.28 (3.80)	-7.21 (3.78)	-7.38 (3.84)	
	N170 peak	-10.71 (4.40)	-11.2 (4.51)	-10.9 (4.41)	-10.86 (4.46)	-11.18 (4.51)	-11.15 (4.51)	-10.86 (4.47)	-11.08 (4.48)	-11 (4.53)	
	EPN	-2.06 (3.21)	-2.1 (3.36)	-2.03 (3.30)	-2.16 (3.45)	-2.2 (3.75)	-2.12 (3.36)	-2.58 (3.10)	-2.52 (3.34)	-2.74 (3.09)	
	LPC	5.06 (3.42)	5.06 (3.38)	5.16 (3.57)	5.61 (3.80)	5.7 (3.96)	5.55 (3.78)	5.55 (3.46)	5.63 (3.56)	5.51 (3.54)	

##### N170: Associated valence

N170 mean amplitudes were not modulated by valence (${\upchi }^{2}$(2) = 2.1, *p* = .350), but there was a main effect of task (${\upchi }^{2}$(1) = 28.72, *p* < .001). Mean amplitudes averaged across valence conditions were significantly more negative in the valence-classification task (−7.16 $\mathrm{\mu V}$; $\beta$_valclass_ = −0.23, *SE* = 0.04, *t* = −5.49) than in the old-new task (−6.69 $\mathrm{\mu V}$). There was no interaction between valence and task (${\upchi }^{2}$(2) = 1.69, *p* = .430). N170 peak amplitudes were not modulated by valence (${\upchi }^{2}$(2) = 1.65, *p* = .437), task (${\chi }^{2}$(1) = 0.21, *p* = .648) or the valence $\times$ task interaction (${\upchi }^{2}$(2) = 0.77, *p* = .681). 

##### Associated emotion

**Fig. 4:**
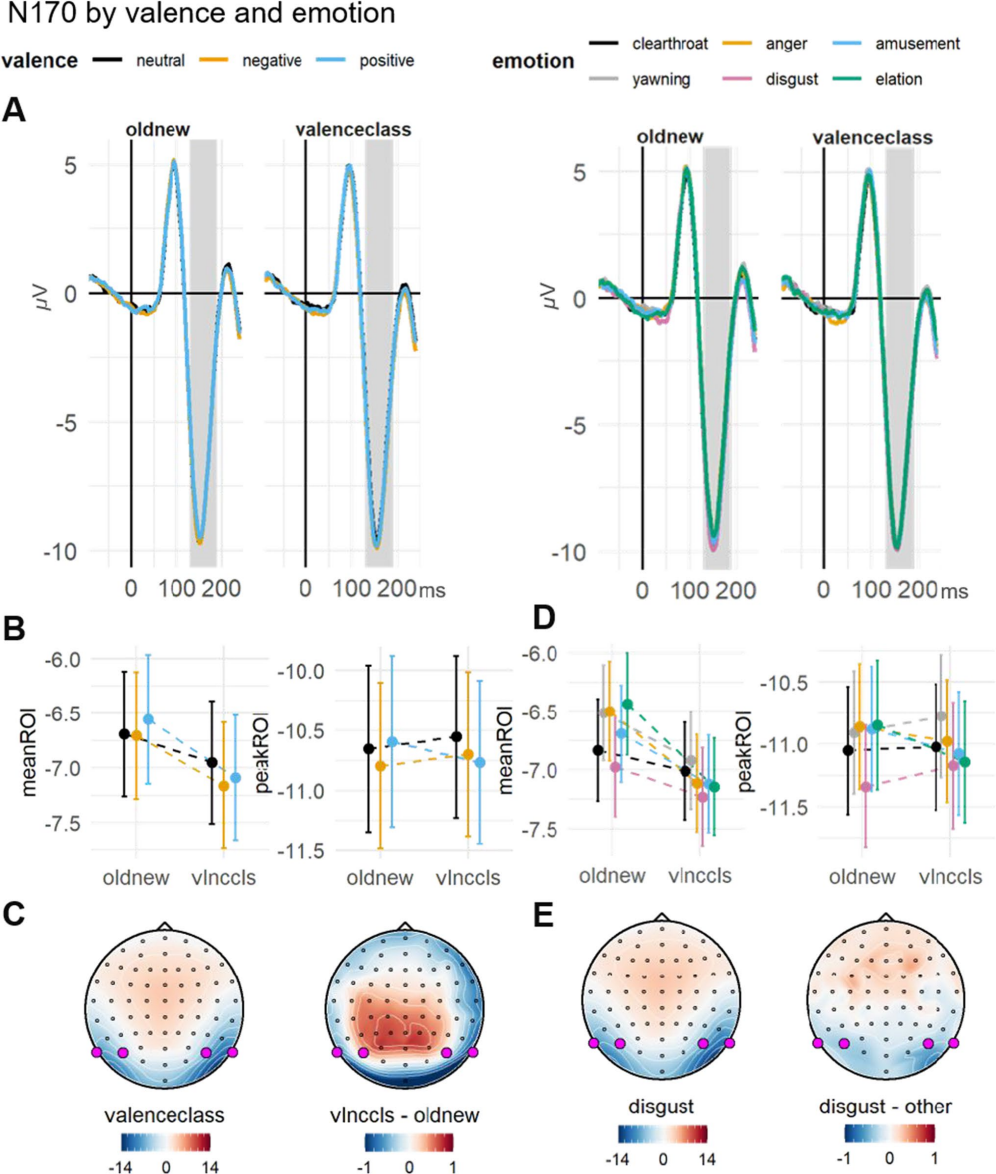
Face-locked N170 by valence and emotion. **A** Grand average ERP time series of the averaged ROI channels (left panel: valence, right panel: emotion). The highlighted area indicates the ROI time window. **B** (valence) and** D** (emotion): Grand-averages of the ROI mean amplitudes (left panel) and peak amplitudes (right panel), contrasted for the implicit and explicit task and all valence/emotion conditions. Error bars indicate ±1 SE of the mean. **C** Topographies of the ERP distribution for faces in the valence-classification task contrasted with the old-new task, averaged across the valence conditions. **E** Topographies of the ERP distribution for faces associated with disgust bursts contrasted with all other emotion conditions, averaged across the tasks. ROI channels are highlighted in pink

Looking at emotion categories separately, N170 mean amplitudes were significantly modulated by emotion (${\upchi }^{2}$(5) = 13.67, *p* = .018), with disgust showing an enhanced negative mean amplitude (−7.21 $\mathrm{\mu V}$; $\beta$_dis_ = −0.28, *SE* = 0.09, *t* = −3.04). Also, in this model, a main effect of task was present (${\upchi }^{2}$(1) = 32.96, *p* < .001) with more negative mean amplitudes for the valence-classification task (−7.17 $\mathrm{\mu V}$; $\beta$_valclass_ = −0.23, *SE* = 0.04, *t* = −5.78). However, there was no interaction between task and emotion present (${\chi }^{2}$(5) = 3.58, *p* = .612). Similar to mean amplitudes, emotion significantly modulated peak amplitudes (${\upchi }^{2}$(5) = 11.58, *p* = .041) with enhanced peak amplitudes for disgust (−11.42 $\mathrm{\mu V}$; $\beta$_dis_ = −0.31, *SE* = 0.10, *t* = −3.01). There was no effect of task (${\upchi }^{2}$(1) = 0.76, *p* = .383) and no interaction between emotion and task (${\upchi }^{2}$(5) = 1.66, *p* = .894) (Fig. 4).

##### EPN: Associated valence

There was a main effect of valence on EPN amplitudes (${\upchi }^{2}$(2) = 10.86, *p* = .004). This was due to enhanced negative amplitudes for negatively (−2.54 $\mathrm{\mu V}$; $\beta$_neg_ = −0.28, *SE* = .09, *t* = −3.23) compared to neutrally (diff_neu-neg_ = 0.47, *p* = .006) and positively (diff_pos-neg_ = 0.37, *p* = .045) associated faces. There was no main effect of task on EPN amplitudes (${\upchi }^{2}$(1) = 0.01, *p* = .925) and no interaction between valence and task (${\upchi }^{2}$(2) = 0.13, *p* = .936).

##### Associated emotion

**Fig. 5:**
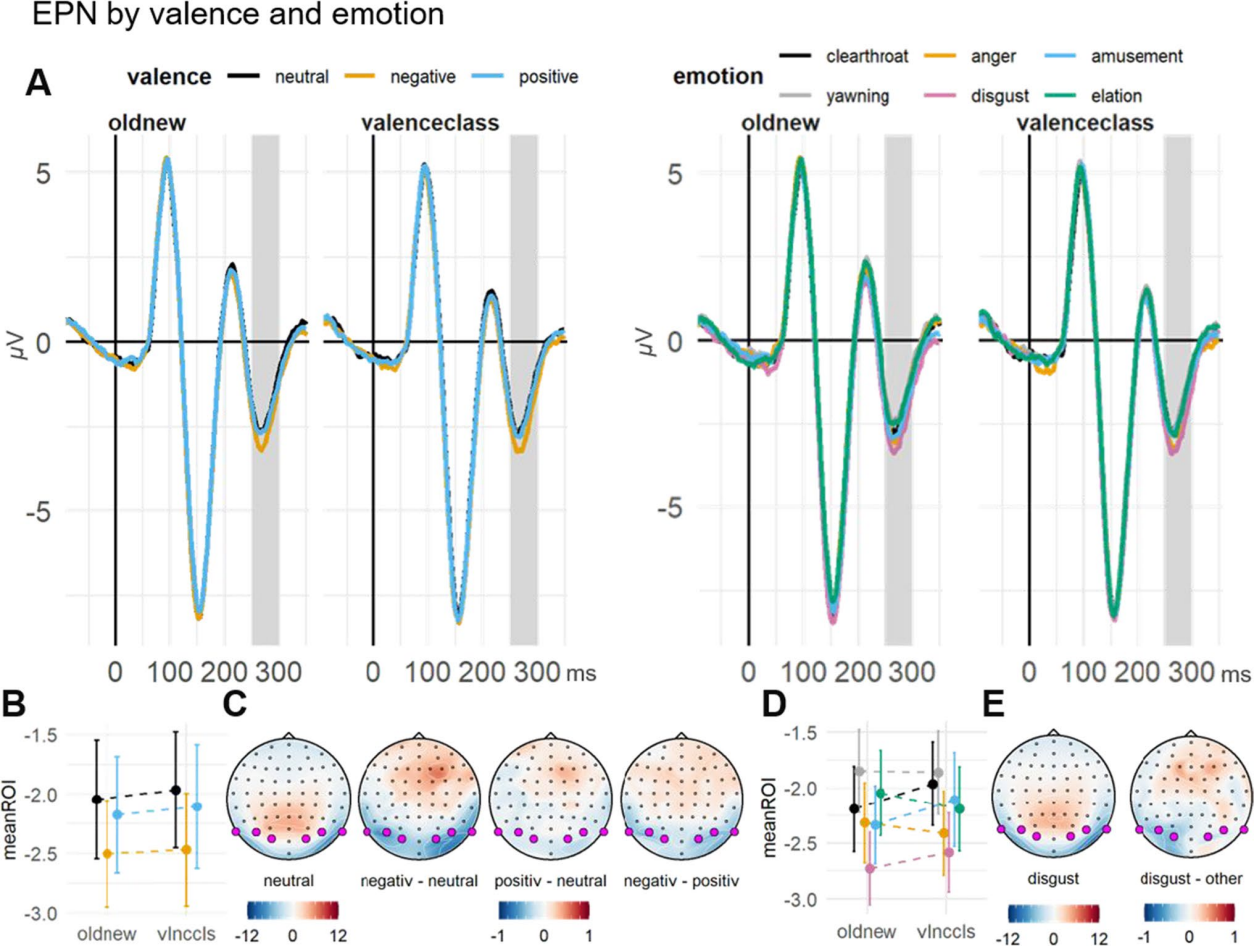
Face-locked EPN by valence and emotion. **A** Grand average ERP time series of the averaged ROI channels for valence (left panel) and emotion (right panel). The highlighted area indicates the ROI time window. **B** (valence) and **D** (emotion): Grand-averages of the ROI mean amplitudes, contrasted for the implicit and explicit task and all valence/emotion conditions. Error bars indicate ±1 SE of the mean. **C** Topographies of the ERP distribution for faces between valence conditions, averaged across the implicit and explicit tasks. **E** Topographies of the ERP distribution for faces associated with disgust bursts contrasted with all other emotion conditions, averaged across the implicit and explicit tasks. ROI channels are highlighted in pink.

Looking at emotion categories separately, there was a main effect of emotion on EPN amplitudes (${\upchi }^{2}$(5) = 21.61, *p* ≤ .001) due to enhanced negative amplitudes for disgust (−2.73 $\mathrm{\mu V}$; $\beta$_dis_ = −0.46, *SE* = 0.12, *t* = −3.79) compared with the neutral categories throat-clearing (diff_dis-clt_ = −0.58, *p* = .031) and yawning (diff_dis-yaw_ = −0.76, *p* ≤ .001) and compared with the positive category elation (diff_dis-el_ = −0.66, *p* = .008), collapsed across tasks. Also in this model, task did not modulate EPN amplitudes (${\upchi }^{2}$(1) = 0.04, *p* = .838) and the emotion $\times$ task interaction (${\upchi }^{2}$(5) = 1.47, *p* = .917) was not significant (Fig. 5).

##### LPC: Associated valence

LPC amplitudes showed no modulation by valence (${\upchi }^{2}$(2) = 2.64, *p* = .268) or task (${\upchi }^{2}$(1) = 1.08, *p* = .298). Although the valence $\times$ task interaction was not significant, (${\upchi }^{2}$(2) = 4.46, *p* = .108), looking at the time course of the component, affectively compared to neutrally associated faces appeared to show a different activation in the valence-classification task. Post-hoc tests showed trends toward a difference between positive and neutral (diff_pos-neu_ = 0.55, *p* = .054), and between negative and neutral categories (diff_neg-neu_ = 0.49, *p* = .098), which were present only in the valence-classification task.

##### Associated emotion

**Fig. 6:**
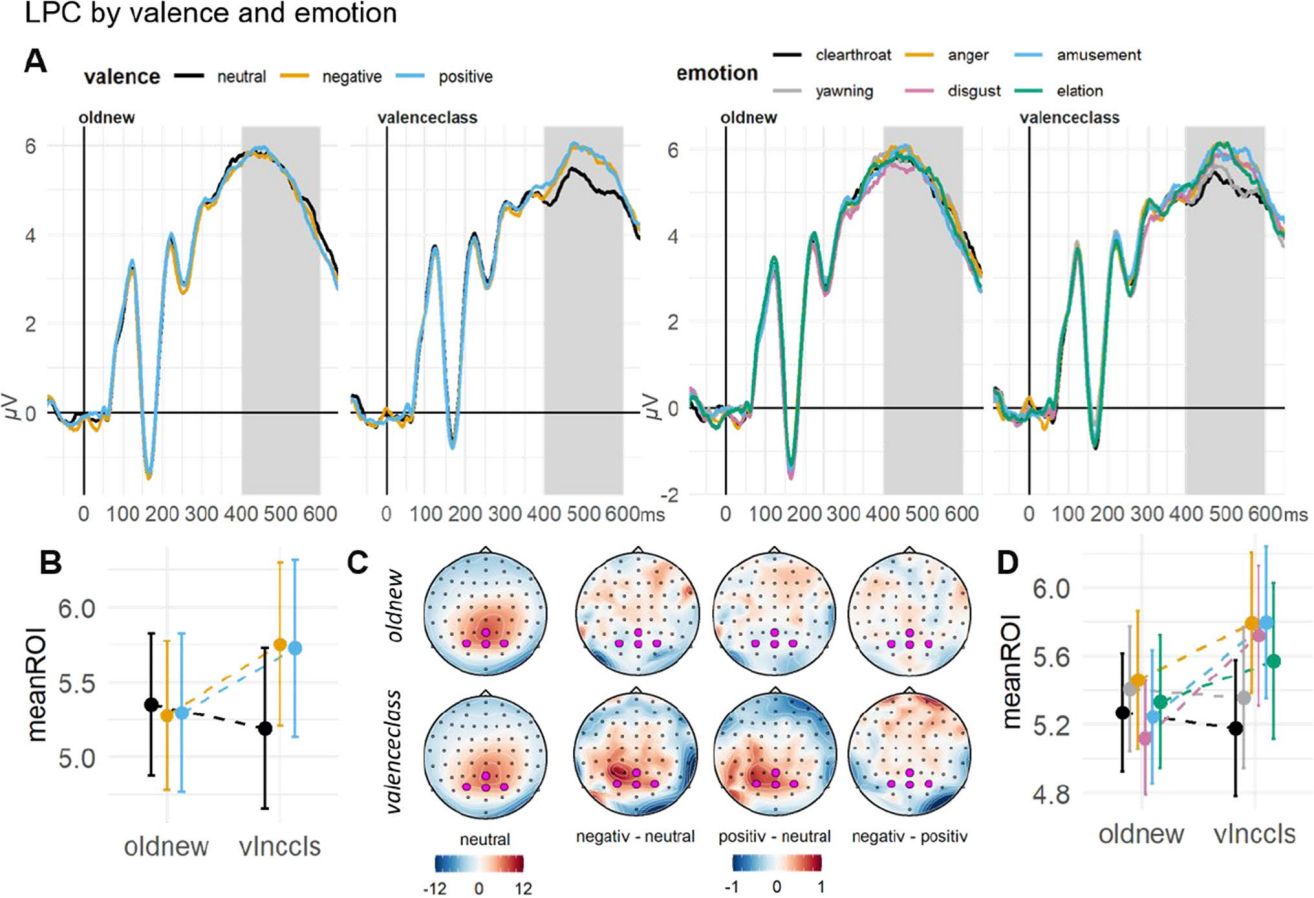
Face-locked LPC by valence and emotion. **A** Grand average ERP time series of the averaged ROI channels for valence (left panel) and emotion (right panel). The highlighted area displays the ROI time window. **B** (valence) and **D** (emotion): Grand averages of the ROI mean amplitudes, contrasted for the implicit and explicit task and all valence/emotion conditions. Error bars indicate ±1 SE of the mean. **C** Topographies of the ERP distribution for faces between valence conditions, separately for the implicit (old-new) and explicit (valence class.) tasks. ROI channels are highlighted in pink.

LPC amplitudes were not significantly explained by individual emotion levels (${\upchi }^{2}$(5) = 5.01, *p* = .414), or task (${\upchi }^{2}$(1) = 2.46, *p* = .117) or an emotion $\times$ task interaction (${\upchi }^{2}$(5) = 6.2, *p* = .287). Descriptively, the neutral categories elicited lower amplitudes in the valence-classification task, but also here, none of the post-hoc contrasts were significant (Fig. 6).

**Fig. 7:**
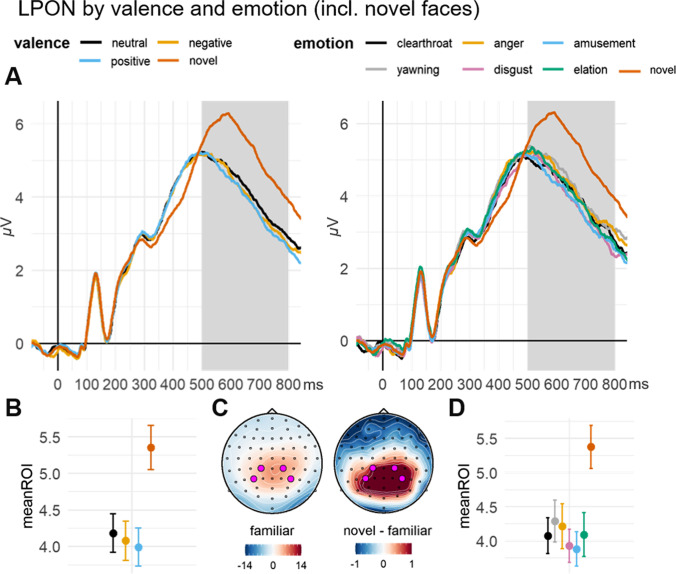
Face-locked LPON by valence and emotion in the old/new task.** A** Grand average ERP time series of the averaged ROI channels for valence (left panel) and emotion (right panel). The highlighted area displays the ROI time window. **B** (valence) and **D** (emotion): Grand-averages of the ROI mean amplitudes, contrasted for all conditions including novel faces. Error bars indicate ±1 SE of the mean. **C** Topographies of the ERP distribution for familiar (i.e., associated) and novel faces. ROI channels are highlighted in pink.

### LPON and FN400

LPON and FN400 mean amplitudes were analyzed only for faces presented in the old-new task. The predictors of the models include a level for novel faces in addition to the valence/emotion levels.

#### **FN400**^3^**:** Associated valence

There was no effect of valence (χ^2^(3) = 2.73, *p* = .436), and thus, no significant difference between associated and novel faces. 

#### Associated emotion

There was a trend for emotion (χ^2^(6) = 10.85, *p* = .093), but none of the post-hoc tests were significant. The largest difference between categories was between yawning and disgust (diff_yaw-dis_ = **−**0.41, *p* = .137).

#### LPON: Associated valence

There was a main effect of valence (χ^2^(3) = 84.87, *p* < .001) with a difference between novel faces and all associated faces (diff_nov-pos_ = 1.36, *p* < .001; diff_nov-neg_ = 1.30, *p* < .001; diff_nov-neu_ = 1.17, *p* < .001). No other difference between valence levels was significant. 

#### Associated emotion

Analogously to the valence model, there was a main effect of emotion (χ^2^(6) = 88.54, *p* < .001) with a significant difference between novel faces and all emotion categories (all *p*-values < .01). None of the other post-hoc contrasts between emotion levels were significant (Fig. 7).

#### Behavioral Outcomes

##### Response times. Associated valence

In line with our hypothesis, responses were slower in the valence-classification task than in the old-new task (diff_valclass-oldnew_ = 212 ms; ${\upchi }^{2}$(1) = 56.42, *p* < .001). There was no main effect of valence (${\upchi }^{2}$(2) = 2.14, *p* = .343), but an interaction between valence and task (${\upchi }^{2}$(2) = 6.55, *p* = .038). In the valence-classification task, neutral trials were descriptively slower than positive (and to a lesser extent also negative) trials, but post-hoc differences were not significant (all *p-*values > .05). 

##### Associated emotion

Due to singularity issues we reduced the model structure to a random intercept model with participant ID. Also in this model, there was a main effect of task (diff_valclass-oldnew_ = 213 ms; ${\upchi }^{2}$(1) = 406.07, *p* < .001). However, neither emotion (${\upchi }^{2}$(5) = 2.68, *p* = .749), nor the interaction between emotion and task was significant (${\upchi }^{2}$(5) = 4.64, *p* = .461). *Old/new task comparison:* When comparing the valence categories and novel stimuli in the old new task, participants responded more slowly to novel faces than to faces known from the learning phase, irrespective of their valence (${\upchi }^{2}$(3) = 19.67, *p* < .001). The largest difference was between positive and novel faces (diff_pos-nov_ = −37 ms, *p* < .001).

##### **Accuracy.** Associated valence

As hypothesized, accuracy was lower in the valence-classification task compared with the old-new task (OR_valclass/oldnew_ = 0.32, ${\upchi }^{2}$(1) = 13.85, *p* < .001). Valence was not significant (${\upchi }^{2}$(2) = 0.13, *p* = .938), and there was no significant interaction between task and valence (${\upchi }^{2}$(2) = 0.56, *p* = .756). 

##### Associated emotion

A model with single emotion levels resulted again in a main effect of task (${\upchi }^{2}$(1) = 14.16, *p* < .001). There was no main effect of emotion (${\upchi }^{2}$(5) = 2.74, *p* = .740) but a significant interaction between task and emotion (${\upchi }^{2}$(5) = 12.72, *p* = .026). Post-hoc tests showed that for all emotion categories but anger (OR = 0.61, *p* = .198) and elation (OR = 0.48, *p* = .051) the valence-classification task had a significantly lower accuracy compared to the old-new task (all *p-*values ≤ .05).

### Likability rating

**Fig. 8:**
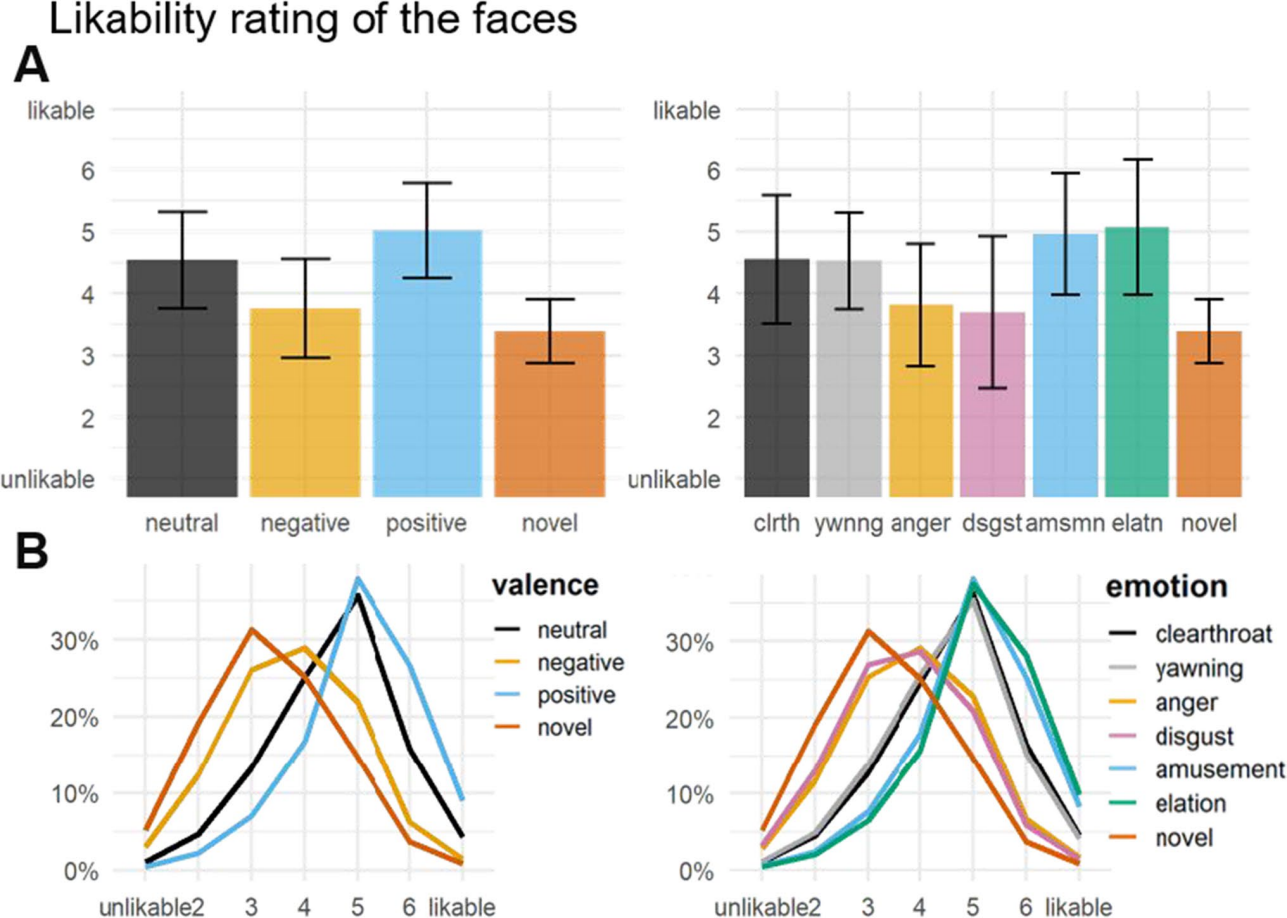
Likability rating. (left panels: by valence, right panels: by emotion). **A** Barplots represent the likability ratings per condition, averaged within and across subjects. Error bars show ±1 SD. **B** Fitted values are the predicted probabilities of the ordinal models.

We ran two (one for valence and one for emotion levels) cumulative linked mixed models to account for the ordinal scale of the likability ratings. Both models included random intercepts for participant and face stimulus. Likelihood ratio tests of both models and a model without a fixed effect indicated that valence significantly explained the variance of the rating data (${\upchi }^{2}$(3) = 96.19, *p* ≤ .001). However, separating emotion categories did not explain the data better than the valence categories (${\upchi }^{2}$(3) = 0.79, *p* = .851). The odds ratios and 95% CI of both models are reported in Table A20 of the Supplementary Information. Mean ratings and model predictions are shown in Fig. 8.

#### Associated valence

Associated valence modulated the likability ratings in line with our hypothesis, with positively associated faces being rated the most likable and negatively associated faces the least likable. With the exception of novel and negatively associated faces, all pairwise differences between valence categories were significant (all *p-*values < .01).

#### Associated emotion

The ordinal model including emotion categories showed a grouping of levels according to the prespecified valence categories. There were no significant differences within valence categories (neutral: throat-clearing and yawning; negative: anger and disgust; positive: amusement and elation). Pairwise comparisons of emotion categories of different valences were significant (all *p-*values < .05), except for throat-clearing and all positive categories, yawning and amusement, and novel and all negative categories.

## Discussion

The present study aimed to investigate memory-based attention effects on the retrieval of valence-based associations in face perception. After having faces associated with affect bursts in an online learning paradigm, we measured short-, mid-, and long-latency ERPs to faces associated with positive, neutral, or negative valence in a valence-implicit and a valence-explicit task. Consistent with our hypotheses and previous research, we found that faces previously associated with affect bursts were not only rated according to the valence of the context but also elicited differential neural responses from faces associated with a neutral context. Moreover, associated effects in late components were strongly affected by task requirements, suggesting that goal-directed attention on specific associated features affected especially later, more elaborate processing of the faces.

The first associated effect was present in the N170. Although the averaged associated valence did not moderate N170 amplitudes, there were differences between individual emotion levels, with an enhanced negative amplitude for disgust-associated faces. A number of studies reported N170 effects for valence-associations (Aguado et al., 2012; Bruchmann et al., 2021; Camfield et al., 2016; Luo et al., 2016; Schellhaas et al., 2020; Schindler et al., 2021; Sperl et al., 2021). Due to its measured spatial overlap with the EPN, the N170 has been suggested to represent a mixture of configural face processing and relevance encoding (Rellecke et al., 2012b). In addition, there was an independent effect of task starting in the N170 time window and extending to a positive-going deflection over the lateral occipito-temporal areas peaking around 200 ms (similar to findings by Itier & Neath-Tavares, 2017, and Schindler et al., 2021). The interpretation of this effect is not straightforward: the visually evoked P2 component has been linked to higher-order configural processing (Latinus & Taylor, 2006), differences in task difficulty (Philiastides, 2006), tasks requiring expertise on subgroups of faces (Stahl et al., 2008), and face typicality (Pell et al., 2022), all of which could be roughly related to deeper processing demands (Banko et al., 2011) of faces in the valence-classification task and differences in processing depth (Itier and Neath-Tavares, 2017). Remarkably, these early task differences did not extend to the EPN time window.

EPN amplitudes were modulated by associated valence, with enhanced amplitudes for the negative compared to the positive and neutral conditions. Several studies reported enhanced neural processing of negatively but not positively associated faces (Luo et al., 2016; Suess et al., 2014; Wieser et al., 2014). This negativity bias also has been shown for threatening facial expressions (Schupp et al., 2004; for a review, see Schindler & Bublatzky, 2020). That negatively associated faces were preferentially processed in our study is remarkable given that affect bursts resemble rather low-intense stimuli. In addition, task neither modulated EPN amplitudes nor did it moderate valence effects, suggesting a rapid and automatic allocation of attention toward negative information related to faces (similar to Baum & Rahman, 2021; Bruchmann et al., 2021; cf. Schindler et al., 2021). Similar to the N170, EPN amplitudes were particularly pronounced for disgust-related faces. Typically, expressions of disgust serve to detect and reject objects that are potentially offensive, toxic, or contaminating to keep oneself safe and healthy (e.g., spoiled food or open wounds). Expressions of disgust directed at us also could serve as social-communicative signals and be interpreted as a risk of social exclusion (Amir et al., 2005; Gan et al., 2022; Judah et al., 2015). Although facial expressions of disapproval, disgust, and anger have been shown to trigger different neural processes (Burklund et al., 2007), auditory expressions of disgust may be perceived as more ambiguous and provide more room for interpretation of social disapproval.

We hypothesized that the attentional focus of the task would particularly affect later processing. Consistent with this hypothesis and previous research on the processing of faces with emotional expressions (for a review, see Schindler & Bublatzky, 2020) and associated faces (Schindler et al., 2021; Bruchmann et al., 2021), associated valence modulations of the LPC were only descriptively present in the valence-explicit task. While early ERPs in the test session showed only effects of negative associations and specifically of disgust-related faces, later processing was modulated by both negatively and positively associated faces, with less strong differences between individual emotion categories. Moreover, positively associated valence was not extinguished but instead triggered by goal-directed memory retrieval, although this was not evident in the valence-implicit task. In our study, LPC modulations were related to the task-relevant goals, while at the same time discriminating between affective and neutral, but not between positive and negative associations. Our results add to findings of previous research reporting LPC effects of positively associated faces (Baum & Rahman, 2021; Hammerschmidt et al., 2018a, b) and other kinds of visual stimuli (Schacht et al., 2012) and also show that positive affect bursts can be cross-modally associated to faces.

P1 amplitudes were not modulated by task and, contrary to our predictions, were not modulated by associated valence. The P1 has been related to the processing of lower-level stimulus properties, and selective attention through sensory gain mechanisms (Hillyard and Anllo-Vento, 1998; Russo, 2003). Several studies have reported a sensitivity of the P1 to valence-based associations (Aguado et al., 2012; Hammerschmidt et al., 2017; Muench et al., 2016; Schacht et al., 2012; Schindler et al., 2022) and even earlier processing (Rehbein et al., 2014; Sperl et al., 2021; Steinberg et al., 2012). However, other studies on associated faces have reported no modulations of the P1 (Hammerschmidt et al., 2018b; Schindler et al., 2021) or have not examined early ERPs (Baum & Rahman, 2021). It is possible that the association with affective vocal stimuli of lower intensity in our study was not sufficient to elicit a differential activation of the P1. Although associated emotional expressions of the face have been shown to modulate P1 amplitudes (Aguado et al., 2012), and comparable effect sizes of cross-modal and within-modal associations have been reported (Hofmann et al., 2010), the variability in learning might have played a more important role. As other studies reported stable associations after very few conditioning trials (Rehbein et al., 2014; Steinberg et al., 2013; Ventura-Bort et al., 2016), it is unclear what drives neural changes at the early processing of conditioned stimuli (e.g., the number of CS-US couplings, the (dis-)similarity between CS, the intensity of the US, the stimulus duration, or the consolidation period, etc.). By including a learning criterion in our study, we ensured associations between the faces and affective bursts. In addition, we included refresher trials between the valence-implicit and valence-explicit tasks to counteract extinction. However, the number of face-voice conditioning trials in the learning phase varied between participants and thus differed from typical conditioning studies. Some participants developed their own strategies and preferred studying the pairs by doing learning checks, which allowed them to see the faces longer and to get feedback on their answer. However, in these trials, not one face but five faces and the voice were presented simultaneously. It is possible that the association of the face and the voice occurred here at a more explicit level and was rather defined by attending to specific facial features rather than by a gradual tuning of sensory discrimination through associative learning.

We included valence ratings of the voices prior to any association with faces. Overall, participants rated the vocal expressions according to our pre-specified valence categories. Interestingly, ratings on their reaction towards the bursts were less extreme than the expression ratings and showed larger interindividual variation. Behavioral performance between valence categories differed only in the learning phase of our study, in which faces with positive bursts were learned faster (similar to reward-associated faces in Hammerschmidt et al., 2017, 2018b; and reward-associated words or symbols, e.g., Bayer et al., 2018; Kulke et al., 2019; Rossi et al., 2017). In contrast, during testing, there was no clear evidence that accuracy and reaction times were affected by associated valence, although descriptively, in the valence-explicit task, responses were slower for the neutral condition than for the positive and negative conditions. Nevertheless, as expected, RTs were shorter and accuracy higher in the old-new task than in the valence-classification task, probably due to the number of choices (two vs. three) and to the required depth of processing (recognition vs. explicit recall). The old-new task might have become more difficult over time due to the repetition of the novel faces. However, the behavioral results suggest that, overall, the valence-classification task was more difficult than the old-new task. If cognitive load alone suppressed ERP effects of associated valence, we would have expected it to occur in the more difficult, i.e., in the valence-classification task. The repeated presentation of non-associated faces, the expectation that they would be repeated, and the relative difficulty of discriminating faces by their inner parts might have prevented typical old/new ERP effects, such as the FN400 and the later parietal effects (Curran & Hancock, 2007; Guillaume & Tiberghien, 2013; Proverbio et al., 2019) in the old-new task.

Likability ratings at the end of the test session were affected by associations with voices expressing positive and negative emotions during the learning (similar to Suess et al., 2014). More specifically, and as we hypothesized, ratings were made according to our pre-specified valence categories, whereas emotion within valence categories did not differ. It is possible that valence, rather than the specific emotion category, altered decisions on likability, although we cannot rule out the possibility that the preceding valence-classification task increased homogenization within valence conditions. Although likability ratings supported the ERP results, in our opinion, the ratings may more closely resemble contingency awareness than true changes in likability and may be biased by the focus on valence differences in the preceding task.

Our novel learning paradigm allowed participants to study the face-voice pairs in a flexible manner, and participants took advantage of this as documented by the learning strategy questionnaire. Despite some variation, participants followed similar self-chosen strategies to memorize the pairs, although we did not provide any hints or recommendations on how to study the face-voice pairs. Participants actively searched for distinct facial features to combine them with what they thought would match the emotional valence of the voice (e.g., the man with tired eyes yawned; the woman with warm brown eyes giggled). As the pairing of the faces and voices was randomized, participants reported taking the features that best distinguished the faces and voices, and some participants even took notes to study. Hence, the type of learning was very different from classical Pavlovian conditioning or instrumental learning, where associations might form more gradually. Remarkably, faces associated with moderately negative bursts elicited distinct neural activation regardless of the task requirements and despite this variability in learning.

One limitation of the study might be that, although we deliberately chose this option, we fixed the order of the tasks in the test session (refresher I, old-new task, refresher II, and the valence-classification task). To ensure that only the valence-classification task would elicit explicit attention to the valence-based associations and to avoid spill-over effects to the valence-implicit task, we set the valence-explicit task at the last position of the experimental part, in which we recorded ERPs. Nevertheless, early effects were similar between tasks, and the valence effects in the LPC occurred only in the valence-classification, i.e., the second task, which should have been more prone to be affected by the extinction of the associations or simply by fatigue.

## Conclusions

The present study provides new evidence that faces cross-modally associated with affective stimuli of both positive and negative valence have the potential to elicit neurophysiological responses similar to those of inherent affective stimuli. During testing, task demands affected later, more effortful processing, whereas earlier processing indicated an automatic discrimination of negative from other information across both tasks. We demonstrated that associations with even mildly negative stimuli, flexibly acquired through our novel learning paradigm, could influence face processing even in a valence-implicit task, suggesting a rapid prioritization of learned negative context as a protection against potential threats (Lundqvist & Öhman, 2005; Öhman et al., 2001), largely independent of goal-directed attention. In addition, positive associations were learned faster and affected later processing, but only in the presence of goal-directed attention toward valence.

### Supplementary Information

Below is the link to the electronic supplementary material.

Supplementary file1 (PDF 312 KB)

## Data Availability

Raw data are not publicly available for privacy reasons (no consent from participants to publish the raw data). The analysis and experimental code of this study is available upon request from the corresponding author, Annika Ziereis. The study was preregistered before data collection (https://osf.io/ts4pb).

## Footnotes

1 Not preregistered.

2 Mnemonic device entailed associating a particular facial feature with a character trait that matches the voice, making up stories about the person, comparing them to somebody they know or giving them names. In some cases, participants wrote their mnemonic devices on paper and used them as a learning aid until they could allocate the faces and voices without it.

3 Two participants (IDs: 30, 7) were excluded from this analysis due to influential observations and Cook**’**s distance >1 in the model with valence as a predictor. To compare results, they were also excluded from the model with separate emotion categories.
