# Supplementary material for: Motivated attention and task relevance in the processing of cross-modally associated faces: Behavioral and electrophysiological evidence
Source: Cogn Affect Behav Neurosci. 2023 Jun 23;23(5):1244–66. doi: 10.3758/s13415-023-01112-5 (PMC10545602; doi:10.3758/s13415-023-01112-5)
Supplement: Supplementary file 1 — Supplementary file1 (PDF 312 KB) [file 13415_2023_1112_MOESM1_ESM.pdf]

Supplentary Information for 'Motivated attention and task relevance in the processing of cross-modally associated faces: Behavioral and electrophysiological evidence'

Annika Ziereis & Anne Schacht

2023-04-05

Contents

Statistical model results 1
Learning phase 1
Test session 2

Exploratory analyses **7**
Correlations between ERPs and likability rating of the faces 8
N1 (voice-locked) 9
P2 (voice-locked) 9
Pupil size 10

References **11**

Correspondence: Goßlerstr. 14, 37073 Goettingen, Germany Email: Annika.Ziereis@uni-goettingen.de

Statistical model results

Learning phase

Table A1. Statistical results for the valence ratings of the affect bursts

	β	SE	t	CI_l	CI_u	LRT:Model	χ^2	df	p
(Intercept)	-0.37	0.08	-4.79	-0.52	-0.22	-	-	-	-
emotionyawning	0.29	0.11	2.68	0.08	0.50				
emotionanger	-1.74	0.15	-11.90	-2.03	-1.45				
emotiondisgust	-1.10	0.13	-8.75	-1.34	-0.85				
emotionamusement	2.17	0.14	15.02	1.89	2.46	emotion	6	0.7	.008
emotionelation	2.22	0.15	14.59	1.93	2.52				
typereaction	0.05	0.11	0.47	-0.16	0.26	type	828.21	23.1	<.001

emotionyawning:typereaction	-0.01	0.15	-0.08	-0.31	0.29	emotion:type	137.78	6.34	<.001
emotionanger:typereaction	0.67	0.19	3.52	0.30	1.04				
emotiondisgust:typereaction	0.20	0.17	1.17	-0.14	0.54				
emotionamusement:typereaction	-0.84	0.19	-4.52	-1.20	-0.48				
emotionelation:typereaction	-1.48	0.19	-7.91	-1.85	-1.12				

Note: beta = model estimate, SE = standard error of the estimate, CI = lower and upper 95% bootstrapped confidence intervals, LRT = Likelihood ratio test

Table A2. Statistical results for accuracy in learning checks during learning

	β	SE	z	CI_l	CI_u	$Stab_{min}$	$Stab_{max}$	LRT:Model	χ^2	df	p
(Intercept)	-0.07	0.17	-0.44	-0.41	0.25	-0.14	0.00	(Intercept)	-	-	-
valence_positive-g.m	0.06	0.14	0.40	-0.25	0.39	0.01	0.13	valence	1.9	2	.387
valence_negative-g.m	-0.18	0.13	-1.36	-0.47	0.12	-0.23	-0.14				
checknr	0.45	0.05	8.49	0.36	0.56	0.42	0.47	checknr	51.09	1	<.001
valence_positive-g.m:checknr	0.05	0.02	2.77	-0.02	0.12	0.03	0.07	valence:checknr	7.95	2	.019
valence_negative-g.m:checknr	-0.02	0.02	-1.15	-0.08	0.05	-0.04	0.00				

Note: beta = model estimate, SE = standard error of the estimate, CI = lower and upper 95% bootstrapped confidence intervals,Stab = estimate ranges leaving out one participant at a time, LRT = Likelihood ratio test

Test session

Table A3. Statistical results for log response times by valence

	β	SE	t	CI_l	CI_u	$Stab_{min}$	$Stab_{max}$	LRT:Model	χ^2	df	p	f^2
(Intercept)	-0.20	0.02	-9.30	-0.25	-0.16	-0.21	-0.20	(Intercept)	-	-	-	-
valence_positive-g.m	-0.01	0.01	-1.37	-0.02	0.00	0.01	0.01	valence	2.14	2	.343	0.05
valence_negative-g.m	0.00	0.01	-0.08	-0.02	0.01	0.00	0.00					
task_valclass-g.m	0.13	0.01	10.99	0.11	0.15	-0.13	-0.12	task	56.42	1	<.001	3.10
valence_positive-g.m:task_valclass-g.m	0.00	0.00	-0.31	-0.01	0.01	-0.01	-0.01	valence:task	6.55	2	.038	0.09
valence_negative-g.m:task_valclass-g.m	-0.01	0.00	-2.06	-0.02	0.00	0.01	0.01					

Note: beta = model estimate, SE = standard error of the estimate, CI = lower and upper 95% bootstrapped confidence intervals, Stab = estimate ranges leaving out participants, LRT = Likelihood ratio test, f2 = Cohen's f2 effect size

Table A4. Statistical results for log response times by emotion

	β	SE	t	CI_l	CI_u	$Stab_{min}$	$Stab_{max}$	LRT:Model	χ^2	df	p	f^2
(Intercept)	-0.20	0.02	-9.37	-0.24	-0.16	-0.21	-0.20	(Intercept)	-	-	-	-
emotion_elation-g.m	0.00	0.01	-0.33	-0.03	0.02	-0.01	0.00	emotion	2.68	5	.749	0.01
emotion_amusement-g.m	-0.01	0.01	-1.06	-0.03	0.01	-0.02	-0.01					
emotion_disgust-g.m	0.00	0.01	-0.13	-0.02	0.02	-0.01	0.00					
emotion_anger-g.m	0.00	0.01	0.07	-0.02	0.02	0.00	0.00					
emotion_yawning-g.m	0.02	0.01	1.37	-0.01	0.04	0.01	0.02					
task_valclass-g.m	0.13	0.01	25.51	0.12	0.14	0.12	0.13	task	406.07	1	<.001	1.52
emotion_elation-g.m:task_valclass-g.m	0.00	0.01	0.24	-0.02	0.02	0.00	0.01	emotion:task	4.64	5	.461	0.01
emotion_amusement-g.m:task_valclass-g.m	-0.01	0.01	-0.51	-0.03	0.02	-0.01	0.00					
emotion_disgust-g.m:task_valclass-g.m	0.00	0.01	-0.01	-0.02	0.02	0.00	0.00					
emotion_anger-g.m:task_valclass-g.m	-0.02	0.01	-1.60	-0.04	0.00	-0.02	-0.01					
emotion_yawning-g.m:task_valclass-g.m	0.02	0.01	1.58	0.00	0.04	0.02	0.02					

Note: beta = model estimate, SE = standard error of the estimate, CI = lower and upper 95% bootstrapped confidence intervals, Stab = estimate ranges leaving out participants, LRT = Likelihood ratio test, f2 = Cohen's f2 effect size

Table A5. Statistical results for log response times in the old-new task (reference: novel)

	β	SE	t	CI_l	CI_u	$Stab_{min}$	$Stab_{max}$	LRT:Model	χ^2	df	p	f^2
(Intercept)	-0.32	0.02	-13.04	-0.37	-0.27	-0.34	-0.32	(Intercept)	-	-	-	-
valence_positive-g.m	-0.02	0.01	-2.35	-0.03	0.00	-0.02	-0.02					
valence_negative-g.m	0.00	0.01	-0.32	-0.02	0.01	0.00	0.00	valence	19.67	3	<.001	0.18
valence_neutral-g.m	-0.01	0.01	-1.71	-0.03	0.00	-0.02	-0.01					

Note: beta = model estimate, SE = standard error of the estimate, CI = lower and upper 95% bootstrapped confidence intervals, Stab = estimate ranges leaving out participants, LRT = Likelihood ratio test, f2 = Cohen's f2 effect size

Table A6. Statistical results for accuracy by valence

	β	SE	z	CI_l	CI_u	$Stab_{min}$	$Stab_{max}$	LRT:Model	χ^2	df	p
(Intercept)	4.73	0.19	25.33	4.38	5.12	4.66	4.78	(Intercept)	-	-	-
valence_positive-g.m	0.05	0.13	0.36	-0.22	0.32	0.00	0.12	valence	0.13	2	.938
valence_negative-g.m	-0.01	0.16	-0.03	-0.32	0.33	-0.06	0.04				
task_valclass-g.m	-0.57	0.15	-3.85	-0.86	-0.30	-0.61	-0.51	task	13.85	1	<.001
valence_positive-g.m:task_valclass-g.m	-0.01	0.09	-0.16	-0.19	0.16	-0.15	0.07	valence:task	0.56	2	.756
valence_negative-g.m:task_valclass-g.m	0.07	0.09	0.73	-0.10	0.26	-0.01	0.16				

Note: beta = model estimate, SE = standard error of the estimate, CI = lower and upper 95% bootstrapped confidence intervals, Stab = estimate ranges leaving out participants, LRT = Likelihood ratio test, f2 = Cohen's f2 effect size

Table A7. Statistical results for accuracy by emotion

	β	SE	z	CI_l	CI_u	$Stab_{min}$	$Stab_{max}$	LRT:Model	χ^2	df	p
(Intercept)	5.00	0.19	25.72	4.66	5.44	4.93	5.07	(Intercept)	-	-	-
emotion_yawning-g.m	-0.26	0.24	-1.05	-0.73	0.26	-0.38	-0.03				
emotion_anger-g.m	0.00	0.23	-0.01	-0.47	0.48	-0.08	0.08				
emotion_disgust-g.m	-0.05	0.24	-0.21	-0.52	0.53	-0.18	0.12	emotion	2.74	5	.740
emotion_amusement-g.m	0.21	0.20	1.05	-0.21	0.72	0.11	0.30				
emotion_elation-g.m	-0.18	0.20	-0.88	-0.62	0.27	-0.29	-0.01				
task_valenceclass-g.m	-0.57	0.14	-4.03	-0.89	-0.30	-0.63	-0.50	task	14.16	1	<.001
emotion_yawning-g.m:task_valenceclass-g.m	-0.15	0.13	-1.18	-0.41	0.09	-0.40	-0.03				
emotion_anger-g.m:task_valenceclass-g.m	0.33	0.13	2.60	0.08	0.60	0.17	0.42				
emotion_disgust-g.m:task_valenceclass-g.m	-0.11	0.13	-0.84	-0.40	0.15	-0.30	0.18	emotion:task	12.72	5	.026
emotion_amusement-g.m:task_valenceclass-g.m	-0.29	0.16	-1.89	-0.69	0.01	-0.43	-0.19				
emotion_elation-g.m:task_valenceclass-g.m	0.21	0.12	1.73	-0.02	0.46	-0.03	0.35				

Note: beta = model estimate, SE = standard error of the estimate, CI = lower and upper 95% bootstrapped confidence intervals, Stab = estimate ranges leaving out participants, LRT = Likelihood ratio test, f2 = Cohen's f2 effect size

Table A8. Statistical results for the P1 mean amplitudes by valence

	β	SE	t	CI_l	CI_u	$Stab_{min}$	$Stab_{max}$	LRT:Model	χ^2	df	p	f^2
(Intercept)	4.04	0.65	6.22	2.76	5.21	3.78	4.30	(Intercept)	-	-	-	-
valence_positive-g.m	-0.02	0.06	-0.23	-0.14	0.12	-0.04	0.01	valence	0.14	2	.931	0.00
valence_negative-g.m	-0.01	0.06	-0.14	-0.15	0.12	-0.03	0.02					
task_valenceclass-g.m	-0.03	0.05	-0.67	-0.12	0.06	-0.05	-0.01	task	0.46	1	.497	0.00
valence_positive-g.m:task_valenceclass-g.m	-0.03	0.06	-0.45	-0.16	0.10	-0.04	0.00	valence:task	0.54	2	.764	0.00
valence_negative-g.m:task_valenceclass-g.m	-0.02	0.06	-0.26	-0.14	0.11	-0.03	0.00					

Note: beta = model estimate, SE = standard error of the estimate, CI = lower and upper 95% bootstrapped confidence intervals, Stab = estimate ranges leaving out participants, LRT = Likelihood ratio test, f2 = Cohen's f2 effect size

Table A9. Statistical results for the P1 mean amplitudes by emotion

	β	SE	t	CI_l	CI_u	$Stab_{min}$	$Stab_{max}$	LRT:Model	χ^2	df	p	f^2
(Intercept)	4.03	0.65	6.20	2.78	5.31	3.77	4.29	(Intercept)	-	-	-	-
emotion_yawning-g.m	0.06	0.10	0.61	-0.13	0.26	0.01	0.10					
emotion_elation-g.m	0.06	0.10	0.58	-0.14	0.28	0.02	0.11					
emotion_disgust-g.m	-0.11	0.10	-1.04	-0.30	0.09	-0.16	-0.07	emotion	2.07	5	.839	0.00
emotion_anger-g.m	0.06	0.10	0.59	-0.14	0.25	0.03	0.09					
emotion_amusement-g.m	-0.05	0.10	-0.45	-0.25	0.15	-0.08	0.00					
task_valenceclass-g.m	-0.02	0.05	-0.51	-0.11	0.07	-0.05	-0.01	task	0.26	1	.607	0.00
emotion_yawning-g.m:task_valenceclass-g.m	0.07	0.10	0.70	-0.15	0.28	0.02	0.11					
emotion_elation-g.m:task_valenceclass-g.m	-0.06	0.10	-0.59	-0.26	0.14	-0.09	0.01					
emotion_disgust-g.m:task_valenceclass-g.m	0.01	0.10	0.13	-0.18	0.23	-0.03	0.04	emotion:task	0.96	5	.965	0.00
emotion_anger-g.m:task_valenceclass-g.m	-0.05	0.10	-0.48	-0.25	0.14	-0.08	-0.01					
emotion_amusement-g.m:task_valenceclass-g.m	0.02	0.10	0.22	-0.16	0.24	-0.01	0.05					

Note: beta = model estimate, SE = standard error of the estimate, CI = lower and upper 95% bootstrapped confidence intervals, Stab = estimate ranges leaving out participants, LRT = Likelihood ratio test, f2 = Cohen's f2 effect size

Table A10. Statistical results for the P1 peak amplitudes by valence

	β	SE	t	CI_l	CI_u	$Stab_{min}$	$Stab_{max}$	LRT:Model	χ^2	df	p	f^2
(Intercept)	6.91	0.63	11.02	5.68	8.18	6.59	7.12	(Intercept)	-	-	-	-
valence_positive-g.m	0.02	0.08	0.23	-0.13	0.17	-0.03	0.04	valence	0.22	2	.896	0.00
valence_negative-g.m	-0.03	0.08	-0.46	-0.18	0.12	-0.07	-0.01					
task_valenceclass-g.m	-0.04	0.05	-0.81	-0.15	0.06	-0.07	-0.02	task	0.67	1	.413	0.00
valence_positive-g.m:task_valenceclass-g.m	0.04	0.08	0.47	-0.11	0.18	0.01	0.06	valence:task	0.91	2	.635	0.00
valence_negative-g.m:task_valenceclass-g.m	0.04	0.08	0.47	-0.11	0.18	0.01	0.05					

Note: beta = model estimate, SE = standard error of the estimate, CI = lower and upper 95% bootstrapped confidence intervals, Stab = estimate ranges leaving out participants, LRT = Likelihood ratio test, f2 = Cohen's f2 effect size

Table A11. Statistical results for the P1 peak amplitudes by emotion

	β	SE	t	CI_l	CI_u	$Stab_{min}$	$Stab_{max}$	LRT:Model	χ^2	df	p	f^2
(Intercept)	7.21	0.63	11.52	6.08	8.50	6.91	7.44	(Intercept)	-	-	-	-
emotion_yawning-g.m	0.21	0.12	1.81	-0.01	0.44	0.17	0.25					
emotion_elation-g.m	0.09	0.12	0.75	-0.14	0.32	0.04	0.13					
emotion_disgust-g.m	-0.14	0.12	-1.20	-0.35	0.09	-0.21	-0.09	emotion	5.33	5	.377	0.01
emotion_anger-g.m	0.00	0.12	0.04	-0.23	0.22	-0.02	0.03					
emotion_amusement-g.m	-0.08	0.12	-0.73	-0.30	0.13	-0.13	-0.04					
task_valenceclass-g.m	-0.05	0.05	-0.98	-0.15	0.04	-0.07	-0.04	task	0.99	1	.319	0.00
emotion_yawning-g.m:task_valenceclass-g.m	-0.04	0.12	-0.32	-0.27	0.18	-0.10	0.00					
emotion_elation-g.m:task_valenceclass-g.m	-0.05	0.12	-0.43	-0.26	0.18	-0.08	0.03					
emotion_disgust-g.m:task_valenceclass-g.m	0.02	0.12	0.21	-0.21	0.25	-0.03	0.05	emotion:task	1.41	5	.923	0.00
emotion_anger-g.m:task_valenceclass-g.m	-0.05	0.12	-0.43	-0.26	0.19	-0.08	-0.01					
emotion_amusement-g.m:task_valenceclass-g.m	0.12	0.12	1.06	-0.11	0.34	0.08	0.16					

Note: beta = model estimate, SE = standard error of the estimate, CI = lower and upper 95% bootstrapped confidence intervals, Stab = estimate ranges leaving out participants, LRT = Likelihood ratio test, f2 = Cohen's f2 effect size

Table A12. Statistical results for the N170 mean amplitudes by valence

	β	SE	t	CI_l	CI_u	$Stab_{min}$	$Stab_{max}$	LRT:Model	χ^2	df	p	f^2
(Intercept)	-6.93	0.59	-11.68	-8.10	-5.74	-7.12	-6.73	(Intercept)	-	-	-	-
valence_positive-g.m	0.04	0.06	0.74	-0.08	0.15	0.02	0.06	valence	2.1	2	.350	0.01
valence_negative-g.m	-0.09	0.06	-1.43	-0.20	0.03	-0.10	-0.06					
task_valenceclass-g.m	-0.23	0.04	-5.49	-0.31	-0.15	-0.25	-0.20	task	28.72	1	<.001	0.15
valence_positive-g.m:task_valenceclass-g.m	-0.04	0.06	-0.66	-0.15	0.07	-0.06	-0.03	valence:task	1.69	2	.430	0.01
valence_negative-g.m:task_valenceclass-g.m	-0.04	0.06	-0.62	-0.15	0.08	-0.05	-0.02					

Note: beta = model estimate, SE = standard error of the estimate, CI = lower and upper 95% bootstrapped confidence intervals, Stab = estimate ranges leaving out participants, LRT = Likelihood ratio test, f2 = Cohen's f2 effect size

Table A13. Statistical results for the N170 mean amplitudes by emotion

	β	SE	t	CI_l	CI_u	$Stab_{min}$	$Stab_{max}$	LRT:Model	χ^2	df	p	f^2
(Intercept)	-6.94	0.59	-11.70	-8.09	-5.76	-7.13	-6.74	(Intercept)	-	-	-	-
emotion_yawning-g.m	0.17	0.09	1.85	0.00	0.37	0.14	0.20					
emotion_elation-g.m	0.13	0.09	1.45	-0.04	0.30	0.10	0.16					
emotion_disgust-g.m	-0.28	0.09	-3.04	-0.44	-0.09	-0.30	-0.24	emotion	13.67	5	.018	0.03
emotion_anger-g.m	0.08	0.09	0.88	-0.10	0.26	0.05	0.11					
emotion_amusement-g.m	-0.03	0.09	-0.37	-0.22	0.16	-0.07	0.00					
task_valenceclass-g.m	-0.23	0.04	-5.78	-0.31	-0.16	-0.26	-0.20	task	32.96	1	<.001	0.08
emotion_yawning-g.m:task_valenceclass-g.m	0.02	0.09	0.20	-0.16	0.19	-0.01	0.05					
emotion_elation-g.m:task_valenceclass-g.m	-0.08	0.09	-0.85	-0.24	0.09	-0.11	-0.05					
emotion_disgust-g.m:task_valenceclass-g.m	0.07	0.09	0.74	-0.11	0.24	0.03	0.11	emotion:task	3.58	5	.612	0.01
emotion_anger-g.m:task_valenceclass-g.m	-0.12	0.09	-1.29	-0.30	0.06	-0.14	-0.08					
emotion_amusement-g.m:task_valenceclass-g.m	0.01	0.09	0.09	-0.17	0.18	-0.02	0.03					

Note: beta = model estimate, SE = standard error of the estimate, CI = lower and upper 95% bootstrapped confidence intervals, Stab = estimate ranges leaving out participants, LRT = Likelihood ratio test, f2 = Cohen's f2 effect size

Table A14. Statistical results for the N170 peak amplitudes by valence

	β	SE	t	CI_l	CI_u	$Stab_{min}$	$Stab_{max}$	LRT:Model	χ^2	df	p	f^2
(Intercept)	-10.79	0.71	-15.26	-12.11	-9.43	-11.04	-10.54	(Intercept)	-	-	-	-
valence_positive-g.m	0.01	0.07	0.17	-0.12	0.15	-0.02	0.03	valence	1.65	2	.437	0.01
valence_negative-g.m	-0.08	0.07	-1.18	-0.21	0.05	-0.11	-0.05					
task_valenceclass-g.m	-0.02	0.05	-0.45	-0.11	0.07	-0.05	0.00	task	0.21	1	.648	0.00
valence_positive-g.m:task_valenceclass-g.m	-0.06	0.07	-0.86	-0.19	0.08	-0.08	-0.05	valence:task	0.77	2	.681	0.00
valence_negative-g.m:task_valenceclass-g.m	0.03	0.07	0.46	-0.11	0.16	0.01	0.05					

Note: beta = model estimate, SE = standard error of the estimate, CI = lower and upper 95% bootstrapped confidence intervals, Stab = estimate ranges leaving out participants, LRT = Likelihood ratio test, f2 = Cohen's f2 effect size

Table A15. Statistical results for the N170 peak amplitudes by emotion

	β	SE	t	CI_l	CI_u	$Stab_{min}$	$Stab_{max}$	LRT:Model	χ^2	df	p	f^2
(Intercept)	-11.11	0.71	-15.76	-12.49	-9.69	-11.35	-10.86	(Intercept)	-	-	-	-
emotion_yawning-g.m	0.17	0.10	1.68	-0.03	0.37	0.14	0.21					
emotion_elation-g.m	0.05	0.10	0.52	-0.14	0.27	0.02	0.09					
emotion_disgust-g.m	-0.31	0.10	-3.01	-0.51	-0.12	-0.35	-0.27	emotion	11.58	5	.041	0.03
emotion_anger-g.m	0.11	0.10	1.04	-0.08	0.32	0.08	0.16					
emotion_amusement-g.m	0.04	0.10	0.36	-0.18	0.24	0.00	0.07					
task_valenceclass-g.m	-0.04	0.05	-0.86	-0.12	0.05	-0.06	-0.02	task	0.76	1	.383	0.00
emotion_yawning-g.m:task_valenceclass-g.m	0.07	0.10	0.72	-0.11	0.28	0.03	0.11					
emotion_elation-g.m:task_valenceclass-g.m	-0.06	0.10	-0.57	-0.26	0.13	-0.10	-0.03					
emotion_disgust-g.m:task_valenceclass-g.m	0.08	0.10	0.76	-0.14	0.28	0.04	0.12	emotion:task	1.66	5	.894	0.00
emotion_anger-g.m:task_valenceclass-g.m	-0.04	0.10	-0.37	-0.24	0.17	-0.08	-0.01					
emotion_amusement-g.m:task_valenceclass-g.m	-0.06	0.10	-0.62	-0.26	0.13	-0.10	-0.03					

Note: beta = model estimate, SE = standard error of the estimate, CI = lower and upper 95% bootstrapped confidence intervals, Stab = estimate ranges leaving out participants, LRT = Likelihood ratio test, f2 = Cohen's f2 effect size

Table A16. Statistical results for the EPN mean amplitudes by valence

	β	SE	t	CI_l	CI_u	$Stab_{min}$	$Stab_{max}$	LRT:Model	χ^2	df	p	f^2
(Intercept)	-2.26	0.49	-4.62	-3.23	-1.33	-2.44	-2.11	(Intercept)	-	-	-	-
valence_positive-g.m	0.09	0.09	1.01	-0.08	0.24	0.06	0.11	valence	10.86	2	.004	0.06
valence_negative-g.m	-0.28	0.09	-3.23	-0.44	-0.12	-0.32	-0.24					
task_valenceclass-g.m	-0.01	0.06	-0.09	-0.12	0.11	-0.04	0.03	task	0.01	1	.925	0.00
valence_positive-g.m:task_valenceclass-g.m	0.02	0.09	0.21	-0.14	0.18	0.00	0.04	valence:task	0.13	2	.936	0.00
valence_negative-g.m:task_valenceclass-g.m	-0.03	0.09	-0.36	-0.19	0.14	-0.05	0.00					

Note: beta = model estimate, SE = standard error of the estimate, CI = lower and upper 95% bootstrapped confidence intervals, Stab = estimate ranges leaving out participants, LRT = Likelihood ratio test, f2 = Cohen's f2 effect size

Table A17. Statistical results for the EPN mean amplitudes by emotion

	β	SE	t	CI_l	CI_u	$Stab_{min}$	$Stab_{max}$	LRT:Model	χ^2	df	p	f^2
(Intercept)	-2.27	0.49	-4.62	-3.28	-1.37	-2.45	-2.12	(Intercept)	-	-	-	-
emotion_yawning-g.m	0.30	0.12	2.51	0.07	0.55	0.25	0.36					
emotion_elation-g.m	0.20	0.12	1.64	-0.04	0.43	0.17	0.24					
emotion_disgust-g.m	-0.46	0.12	-3.79	-0.69	-0.23	-0.50	-0.40	emotion	21.61	5	<.001	0.05
emotion_anger-g.m	-0.15	0.12	-1.25	-0.39	0.10	-0.19	-0.11					
emotion_amusement-g.m	-0.01	0.12	-0.12	-0.24	0.22	-0.05	0.02					
task_valenceclass-g.m	-0.01	0.05	-0.20	-0.12	0.09	-0.04	0.03	task	0.04	1	.838	0.00
emotion_yawning-g.m:task_valenceclass-g.m	-0.05	0.12	-0.42	-0.28	0.19	-0.09	-0.01					
emotion_elation-g.m:task_valenceclass-g.m	-0.04	0.12	-0.31	-0.28	0.21	-0.08	0.00					
emotion_disgust-g.m:task_valenceclass-g.m	0.01	0.12	0.05	-0.24	0.23	-0.03	0.03	emotion:task	1.47	5	.917	0.00
emotion_anger-g.m:task_valenceclass-g.m	-0.08	0.12	-0.68	-0.32	0.15	-0.12	-0.04					
emotion_amusement-g.m:task_valenceclass-g.m	0.10	0.12	0.84	-0.12	0.34	0.06	0.14					

Note: beta = model estimate, SE = standard error of the estimate, CI = lower and upper 95% bootstrapped confidence intervals, Stab = estimate ranges leaving out participants, LRT = Likelihood ratio test, f2 = Cohen's f2 effect size

Table A18. Statistical results for the LPC mean amplitudes by valence

	β	SE	t	CI_l	CI_u	$Stab_{min}$	$Stab_{max}$	LRT:Model	χ^2	df	p	f^2
(Intercept)	5.34	0.52	10.30	4.33	6.29	5.00	5.51	(Intercept)	-	-	-	-
valence_positive-g.m	0.09	0.09	0.98	-0.10	0.27	0.05	0.12	valence	2.64	2	.268	0.01
valence_negative-g.m	0.06	0.09	0.61	-0.11	0.24	0.04	0.08					
task_valenceclass-g.m	0.07	0.07	1.03	-0.06	0.19	0.04	0.12	task	1.08	1	.298	0.01
valence_positive-g.m:task_valenceclass-g.m	0.11	0.09	1.16	-0.08	0.30	0.08	0.14	valence:task	4.46	2	.108	0.02
valence_negative-g.m:task_valenceclass-g.m	0.09	0.09	0.93	-0.10	0.27	0.06	0.11					

Note: beta = model estimate, SE = standard error of the estimate, CI = lower and upper 95% bootstrapped confidence intervals, Stab = estimate ranges leaving out participants, LRT = Likelihood ratio test, f2 = Cohen's f2 effect size

Table A19. Statistical results for the LPC mean amplitudes by emotion

	β	SE	t	CI_l	CI_u	$Stab_{min}$	$Stab_{max}$	LRT:Model	χ^2	df	p	f^2
(Intercept)	5.35	0.52	10.31	4.40	6.35	5.02	5.53	(Intercept)	-	-	-	-
emotion_yawning-g.m	-0.09	0.12	-0.72	-0.32	0.17	-0.14	-0.06					
emotion_elation-g.m	0.06	0.12	0.52	-0.18	0.30	0.00	0.11					
emotion_disgust-g.m	-0.05	0.12	-0.37	-0.28	0.21	-0.09	0.01	emotion	5.01	5	.414	0.01
emotion_anger-g.m	0.16	0.12	1.33	-0.07	0.42	0.12	0.20					
emotion_amusement-g.m	0.10	0.12	0.83	-0.13	0.35	0.06	0.14					
task_valenceclass-g.m	0.09	0.05	1.55	-0.01	0.19	0.05	0.13	task	2.46	1	.117	0.01
emotion_yawning-g.m:task_valenceclass-g.m	-0.18	0.12	-1.49	-0.41	0.07	-0.21	-0.16					
emotion_elation-g.m:task_valenceclass-g.m	0.05	0.12	0.39	-0.19	0.31	0.01	0.07					
emotion_disgust-g.m:task_valenceclass-g.m	0.12	0.12	1.00	-0.12	0.35	0.09	0.16	emotion:task	6.2	5	.287	0.01
emotion_anger-g.m:task_valenceclass-g.m	0.03	0.12	0.27	-0.21	0.26	0.00	0.07					
emotion_amusement-g.m:task_valenceclass-g.m	0.16	0.12	1.29	-0.08	0.40	0.13	0.19					

Note: beta = model estimate, SE = standard error of the estimate, CI = lower and upper 95% bootstrapped confidence intervals, Stab = estimate ranges leaving out participants, LRT = Likelihood ratio test, f2 = Cohen's f2 effect size

Table A20. Statistical results of the ordinal mixed model for the likability rating

	OR_val	CI_val	OR_emo	CI_emo
neutral-g.m	1.64	[1.31;2.16]		
negative-g.m	0.55	[0.4;0.69]		
positive-g.m	3.62	[2.87;5.16]		
clearthroat-g.m			1.47	[0.95;2.32]
yawning-g.m			1.32	[0.94;1.91]
anger-g.m			0.49	[0.31;0.71]
disgust-g.m			0.43	[0.26;0.63]
amusement-g.m			2.78	[1.89;4.47]
elation-g.m			3.37	[2.36;5.44]
1|2	0.02	[0.01;0.02]	0.01	[0.01;0.02]
2|3	0.10	[0.07;0.11]	0.08	[0.06;0.09]
3|4	0.39	[0.3;0.45]	0.33	[0.25;0.38]
4|5	1.30	[1.1;1.56]	1.10	[0.92;1.33]
5|6	6.52	[5.87;9.18]	5.51	[4.9;7.64]
6|7	36.57	[31.43;67.45]	30.99	[25.93;55.15]

Note: Model estimates and threshold coefficients in Odds Ratios (OR) of the ordinal models (left: valence model, right: emotion model). Brackets indicate the 95% asymptotic confidence intervals of the OR.

Exploratory analyses

Table A21. Statistical results for the FN400 mean amplitudes by valence

	β	SE	t	CI_l	CI_u	$Stab_{min}$	$Stab_{max}$	LRT:Model	χ^2	df	p	f^2
(Intercept)	-0.86	0.28	-3.06	-1.42	-0.31	-0.96	-0.72	(Intercept)	-	-	-	-
valence_novel-g.m	0.00	0.07	0.00	-0.14	0.14	-0.04	0.02					
valence_positive-g.m	0.01	0.07	0.11	-0.13	0.14	-0.03	0.03	valence	2.73	3	.436	0.02
valence_negative-g.m	0.09	0.07	1.28	-0.04	0.22	0.07	0.12					

Note: beta = model estimate, SE = standard error of the estimate, CI = lower and upper 95% bootstrapped confidence intervals, Stab = estimate ranges leaving out participants, LRT = Likelihood ratio test, f2 = Cohen's f2 effect size

Table A22. Statistical results for the FN400 mean amplitudes by emotion

	β	SE	t	CI_l	CI_u	$Stab_{min}$	$Stab_{max}$	LRT:Model	χ^2	df	p	f^2
(Intercept)	-0.87	0.28	-3.14	-1.41	-0.39	-0.97	-0.73	(Intercept)	-	-	-	-
emotion_yawning-g.m	-0.20	0.10	-2.01	-0.40	-0.01	-0.22	-0.17					
emotion_novel-g.m	0.02	0.10	0.16	-0.18	0.21	-0.03	0.04					
emotion_elation-g.m	-0.13	0.10	-1.34	-0.32	0.05	-0.16	-0.09					
emotion_disgust-g.m	0.21	0.10	2.15	0.03	0.41	0.18	0.26	emotion	10.85	6	.093	0.05
emotion_anger-g.m	-0.03	0.10	-0.31	-0.23	0.15	-0.07	0.01					
emotion_amusement-g.m	0.14	0.10	1.42	-0.06	0.34	0.09	0.18					

Note: beta = model estimate, SE = standard error of the estimate, CI = lower and upper 95% bootstrapped confidence intervals, Stab = estimate ranges leaving out participants, LRT = Likelihood ratio test, f2 = Cohen's f2 effect size

Table A23. Statistical results for the LPON mean amplitudes by valence

	β	SE	t	CI_l	CI_u	$Stab_{min}$	$Stab_{max}$	LRT:Model	χ^2	df	p	f^2
(Intercept)	4.46	0.28	15.76	3.92	4.98	4.34	4.55	(Intercept)	-	-	-	-
valence_novel-g.m	0.96	0.09	10.99	0.78	1.13	0.91	1.00					
valence_positive-g.m	-0.40	0.09	-4.59	-0.56	-0.22	-0.43	-0.37	valence	84.87	3	<.001	1.11
valence_negative-g.m	-0.34	0.09	-3.96	-0.52	-0.18	-0.39	-0.32					

Note: beta = model estimate, SE = standard error of the estimate, CI = lower and upper 95% bootstrapped confidence intervals, Stab = estimate ranges leaving out participants, LRT = Likelihood ratio test, f2 = Cohen's f2 effect size

Table A24. Statistical results for the LPON mean amplitudes by emotion

	β	SE	t	CI_l	CI_u	$Stab_{min}$	$Stab_{max}$	LRT:Model	χ^2	df	p	f^2
(Intercept)	4.33	0.28	15.44	3.73	4.86	4.21	4.42	(Intercept)	-	-	-	-
emotion_yawning-g.m	0.05	0.11	0.40	-0.17	0.27	0.01	0.10					
emotion_novel-g.m	1.12	0.11	9.80	0.91	1.34	1.07	1.16					
emotion_elation-g.m	-0.14	0.11	-1.20	-0.35	0.08	-0.19	-0.10	emotion	88.54	6	<.001	0.47
emotion_disgust-g.m	-0.35	0.11	-3.05	-0.57	-0.13	-0.38	-0.31					
emotion_anger-g.m	-0.07	0.11	-0.57	-0.27	0.16	-0.12	-0.03					
emotion_amusement-g.m	-0.39	0.11	-3.46	-0.62	-0.16	-0.44	-0.35					

Note: beta = model estimate, SE = standard error of the estimate, CI = lower and upper 95% bootstrapped confidence intervals, Stab = estimate ranges leaving out participants, LRT = Likelihood ratio test, f2 = Cohen's f2 effect size

Correlations between ERPs and likability rating of the faces

Table A25. Spearman's rank correlation (rho) between the likability rating and single-average ERP component separately by valence and task

		Old-New task		Valence-classification task	
Component	Valence	rho	p	rho	p
P1	positive:	0.13	.045	0.11	.128
P1	negative:	0.08	.197	0.08	.211
	neutral:	0.13	.052	0.1	.072
	positive:	0.08	.178	0.08	.244
N170	negative:	0.03	.553	0.04	.637
	neutral:	-0.15	.131	-0.13	.173
	positive:	0.13	.081	0.06	.345
EPN	negative:	0.01	.733	0.04	.421
	neutral:	-0.03	.642	-0.05	.717
	positive:	0.07	.510	0.1	.216
LPC	negative:	-0.04	.465	-0.08	.788
	neutral:	0.11	.316	0.15	.024
	positive:	-0.12	.147		
FN400	negative:	0.02	.663		
	neutral:	-0.05	.562		
	positive:	0.11	.254		
LPON	negative:	0.05	.314		
	neutral:	0.12	.106		

Note:
Correlations on single averaged ERPs and likeability rating per associated face (i.e., averaged per emotion but not valence category) were performed for each pre-specified valence category separately.

N1 (voice-locked)

N1 amplitudes were not significantly modulated by valence ($\chi^2(2) = 0.5$, $p = .779$).

Table A26. Statistical results for the (auditory) N1 mean amplitudes by valence

	β	SE	t	CI_l	CI_u	$Stab_{min}$	$Stab_{max}$	LRT:Model	χ^2	df	p	f^2
(Intercept)	1.11	0.13	8.64	0.85	1.36	1.07	1.16	(Intercept)	-	-	-	-
valence_positive-g.m	-0.04	0.09	-0.44	-0.21	0.13	-0.06	-0.01	valence	0.5	2	.779	0.01
valence_negative-g.m	0.06	0.09	0.69	-0.13	0.23	0.03	0.09					

Note: beta = model estimate, SE = standard error of the estimate, CI = lower and upper 95% bootstrapped confidence intervals, Stab = estimate ranges leaving out participants, LRT = Likelihood ratio test, f2 = Cohen's f2 effect size

Similarly, N1 amplitudes were not significantly modulated by emotion ($\chi^2(5) = 3.65$, $p = .601$).

Table A27. Statistical results for the (auditory) N1 mean amplitudes by emotion

	β	SE	t	CI_l	CI_u	$Stab_{min}$	$Stab_{max}$	LRT:Model	χ^2	df	p	f^2
(Intercept)	1.11	0.13	8.62	0.86	1.35	1.08	1.16	(Intercept)	-	-	-	-
emotion_yawning-g.m	-0.19	0.13	-1.40	-0.46	0.08	-0.23	-0.12					
emotion_elation-g.m	-0.06	0.13	-0.44	-0.33	0.20	-0.09	0.00					
emotion_disgust-g.m	0.13	0.13	0.95	-0.15	0.40	0.08	0.17	emotion	3.65	5	.601	0.02
emotion_anger-g.m	-0.02	0.13	-0.17	-0.31	0.23	-0.08	0.03					
emotion_amusement-g.m	-0.01	0.13	-0.06	-0.27	0.23	-0.04	0.03					

Note: beta = model estimate, SE = standard error of the estimate, CI = lower and upper 95% bootstrapped confidence intervals, Stab = estimate ranges leaving out participants, LRT = Likelihood ratio test, f2 = Cohen's f2 effect size

P2 (voice-locked)

Due to influential observations (Cook's distance > 1) of one participant in the valence model, this participant was also excluded of the emotion model to allow comparisons of the results. P2 amplitudes were not significantly modulated by valence ($\chi^2(2) = 2.79$, $p = .247$).

Table A28. Statistical results for the (auditory) P2 mean amplitudes by valence

	β	SE	t	CI_l	CI_u	$Stab_{min}$	$Stab_{max}$	LRT:Model	χ^2	df	p	f^2
(Intercept)	1.84	0.15	12.24	1.55	2.15	1.79	1.89	(Intercept)	-	-	-	-
valence_positive-g.m	-0.12	0.08	-1.45	-0.28	0.04	-0.15	-0.09	valence	2.79	2	.247	0.04
valence_negative-g.m	0.12	0.08	1.43	-0.04	0.28	0.09	0.15					

Note: beta = model estimate, SE = standard error of the estimate, CI = lower and upper 95% bootstrapped confidence intervals, Stab = estimate ranges leaving out participants, LRT = Likelihood ratio test, f2 = Cohen's f2 effect size

However, when including emotion categories separately, there was a main effect of emotion on P2 amplitudes ($\chi^2(5) = 13.78$, $p = .017$). Post-hoc tests showed that there was a significant difference between the two neutral pre-specified emotion categories, yawning and throat-clearing ($diff_{\text{yaw-clt}}$ = -0.67, $p = .017$). None of the other pairwise comparisons were significant.

Table A29. Statistical results for the (auditory) P2 mean amplitudes by emotion

	β	SE	t	CI_l	CI_u	$Stab_{min}$	$Stab_{max}$	LRT:Model	χ^2	df	p	f^2
(Intercept)	1.85	0.15	12.33	1.58	2.16	1.80	1.90	(Intercept)	-	-	-	-
emotion_yawning-g.m	-0.32	0.13	-2.42	-0.56	-0.06	-0.37	-0.28					
emotion_elation-g.m	-0.16	0.13	-1.24	-0.41	0.09	-0.20	-0.12					
emotion_disgust-g.m	0.03	0.13	0.22	-0.24	0.27	-0.01	0.08	emotion	13.78	5	.017	0.07
emotion_anger-g.m	0.17	0.13	1.27	-0.08	0.43	0.12	0.21					
emotion_amusement-g.m	-0.07	0.13	-0.53	-0.33	0.20	-0.10	-0.03					

Note: beta = model estimate, SE = standard error of the estimate, CI = lower and upper 95% bootstrapped confidence intervals, Stab = estimate ranges leaving out participants, LRT = Likelihood ratio test, f2 = Cohen's f2 effect size

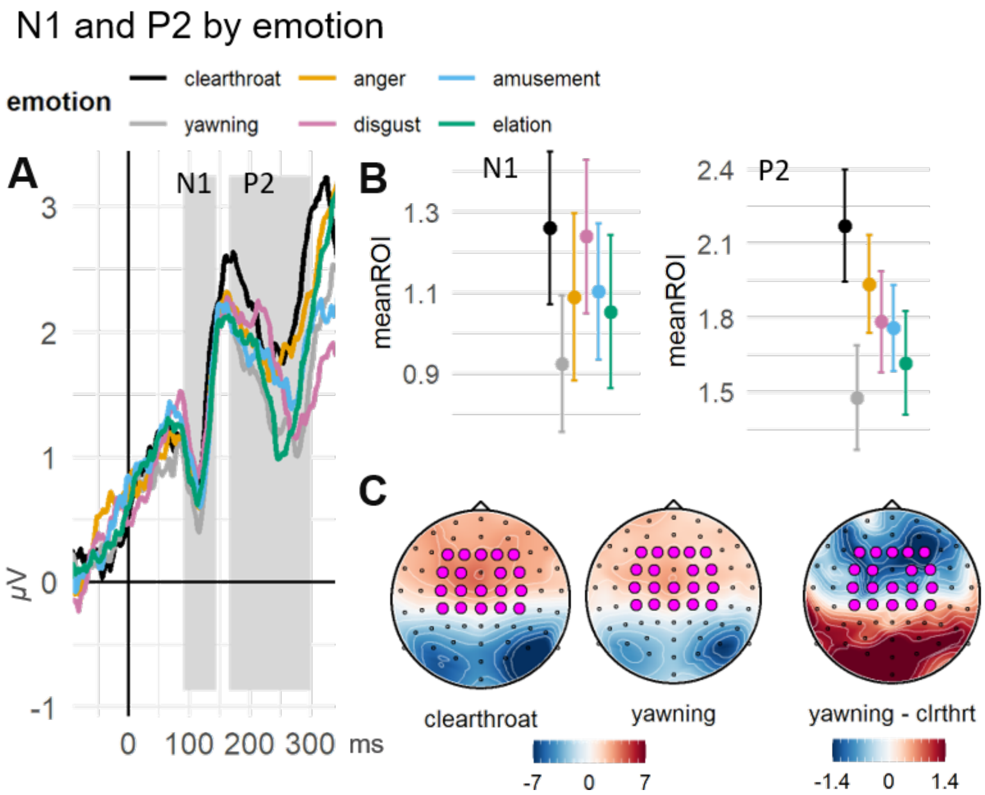

Figure A2. Voice-locked N1 and P2 by emotion (refresher trials). **A.** Grand average ERP time series of the averaged ROI channels. The highlighted area displays the ROI time window. **B.** Grand-averages of the ROI mean amplitudes of the N1 (left panel) and P2 (right panel), contrasted for all emotion conditions. Errorbars indicate +/- 1 SE of the mean. **C.** Topographies of the ERP distribution of the P2 for the two neutral emotion categories 'throatclearing' and 'yawning' and their difference. ROI channels are highlighted in pink.

Pupil size

We used a desktop-mounted eye-tracker (EyeLink 1000 CL 1 - AAD01, SR Research, software version 4.56) to record the pupil size binocularly in pixels with a sampling rate of 500 Hz. Preprocessing was done in (R Core Team 2020) and oriented to guidelines proposed by Kret and Sjak-Shie (2018). The continuous data was epoched around the onset of the face stimulus, and the mean of a 200 ms baseline time window was subtracted. Blinks were classified when samples of both eyes were missing. Other invalid, isolated, or implausible samples due to fast changes or deviations from a smoothed trend line were rejected. Additionally, trials were segmented into 60 bins, and outlier samples per bin (>3 *SD* from the bin mean) were excluded.

If a trial contained less than 75% of valid samples, the whole trial was rejected. A smoothing 4 Hz filter was applied before trials were averaged by condition and participant. Pupil responses primarily served to identify eye blinks. Additionally, we tested valence differences in the refresher trials as a proxy for arousal without a preregistered hypothesis.

We analyzed pupil size during refresher trials to have an additional indicator for arousal of the face-voice pairs. However, model diagnostics indicated that several observations had a strong impact on model results, which hence might not be robust. Removing influential observations (Cook's distance >1) in several rounds led to other observations being classified as influential down to a remaining sample size of 30 participants[1]. However, there was no significant modulation of pupil size by valence for both, the full sample set ($\chi^2(2) = 3.19$, $p = .203$) and the subset of 30 participants ($\chi^2(2) = 2.02$, $p = .365$).

Table A30. Statistical results for pupil diameter during refresher trials

	β	SE	t	CI_l	CI_u	$Stab_{min}$	$Stab_{max}$	LRT:Model	χ^2	df	p	f^2
(Intercept)	-26.95	6.06	-4.45	-39.11	-15.14	-29.25	-25.49	(Intercept)	-	-	-	-
valence_positive-g.m	-0.87	3.64	-0.24	-8.04	6.19	-2.30	0.40	valence	3.19	2	.203	0.04
valence_negative-g.m	5.99	3.64	1.65	-1.46	13.10	4.22	7.26					

Note: beta = model estimate, SE = standard error of the estimate, CI = lower and upper 95% bootstrapped confidence intervals, Stab = estimate ranges leaving out participants, LRT = Likelihood ratio test, f2 = Cohen's f2 effect size

References

Kret, Mariska E., and Elio E. Sjak-Shie. 2018. "Preprocessing Pupil Size Data: Guidelines and Code." *Behavior Research Methods* 51 (3): 1336–42. https://doi.org/10.3758/s13428-018-1075-y.

R Core Team. 2020. *R: A Language and Environment for Statistical Computing.* Vienna, Austria: R Foundation for Statistical Computing. https://www.R-project.org/.

[1] excluded participant IDs were: 4, 5, 9, 13, 15, 16, 17, 48, 53, 56
